# Bioenzymatic single-cell microencapsulation for enhanced stem Cell therapy

**DOI:** 10.1016/j.bioactmat.2026.01.017

**Published:** 2026-01-21

**Authors:** Leyan Xuan, Tingting Lu, Yingying Hou, Yuguang Zhu, Bingbing Zhan, Jialin Wu, Kaixiang Li, Jiachu Huang, Huaibin Wang, Ziyang Liu, Wenqi Xiao, Junjie Cai, Lijie Chen, Jie Wang, Jie Guo, Shufang Wang, Chenrui An, Xiyong Yu, Wei Fu, Guosheng Tang

**Affiliations:** a Guangzhou Municipal and Guangdong Provincial Key Laboratory of Molecular Target & Clinical Pharmacology, the NMPA and State Key Laboratory of Respiratory Disease, School of Pharmaceutical Sciences, Guangzhou Medical University, Guangzhou, 511436, China; b Institute of Pediatric Translational Medicine, Shanghai Institute of Pediatric Congenital Heart Disease, Shanghai Children's Medical Center, Shanghai Jiao Tong University School of Medicine, Shanghai, 200127, China; c Wuhan Children's Hospital (Wuhan Maternal and Child Healthcare Hospital), Tongji Medical College, Huazhong University of Science & Technology, 430074, China; d Department of Pediatric Cardiothoracic Surgery, Shanghai Children's Medical Center, Shanghai Jiao Tong University School of Medicine, Shanghai, 200127, China; e Key Laboratory of Bioactive Materials for the Ministry of Education, College of Life Sciences, Nankai University, Tianjin, 300071, China; f Division of Engineering in Medicine, Department of Medicine, Brigham and Women's Hospital, Harvard Medical School, Cambridge, MA, 02139, USA; g Department of Obstetrics and Gynecology, Guangdong Provincial Key Laboratory of Major Obstetric Diseases, Guangdong Provincial Clinical Research Center for Obstetrics and Gynecology, Guangdong-Hong Kong-Macao Greater Bay Area Higher Education Joint Laboratory of Maternal-Fetal Medicine, The Third Affiliated Hospital, Guangzhou Medical University, Guangzhou, 510150, China

**Keywords:** Bioenzymatic strategy, Single-cell microgel, Stem Cell therapy, Biomedical engineering

## Abstract

Cell therapy has achieved a critical breakthrough through single-cell microgel technology. This miniaturized encapsulation platform enables precise microenvironment recapitulation, efficient targeted delivery, and tunable pericellular matrix control. Nevertheless, prevailing microfluidic and surface chemical engineering methodologies confront fundamental challenges in preserving cell viability and functionality. Here, we establish a simple and bioenzymatic strategy for fabricating single-cell microgels, using microbial transglutaminase adsorption. This surfactant- and oil-free approach, without surface modification, permits universal, high-viability encapsulation of diverse cell types and biomaterials. We achieve 100 % encapsulation efficiency and robust mechanical protection. Therapeutic efficacy was assessed in myocardial infarction (MI) and pulmonary fibrosis (PF) models. In MI, microgel-encapsulated MSCs (MSC SCMs) significantly improved in vivo retention and survival, exhibiting superior tissue regeneration and cardiac function. In bleomycin-induced PF, TNF-α-loaded MSC SCMs potentiated MMP-13 secretion, achieving enhanced respiratory function and attenuated fibrotic lesions. This robust and universally applicable platform thus for advanced cell therapies, overcomes limitations in encapsulation while demonstrating potent therapeutic efficacy across disease models.

## Highlights

• Bioenzymatic single-cell microgel encapsulation via microbial transglutaminase.
• Surfactant- and oil-free method with 100 % encapsulation efficiency.
• Universal compatibility with diverse cell types and biomaterials.
• Enhances cell retention and survival in myocardial infarction models.
• Improves respiratory function and reduces fibrosis in pulmonary fibrosis.

## Graphical abstract

**Figure:**
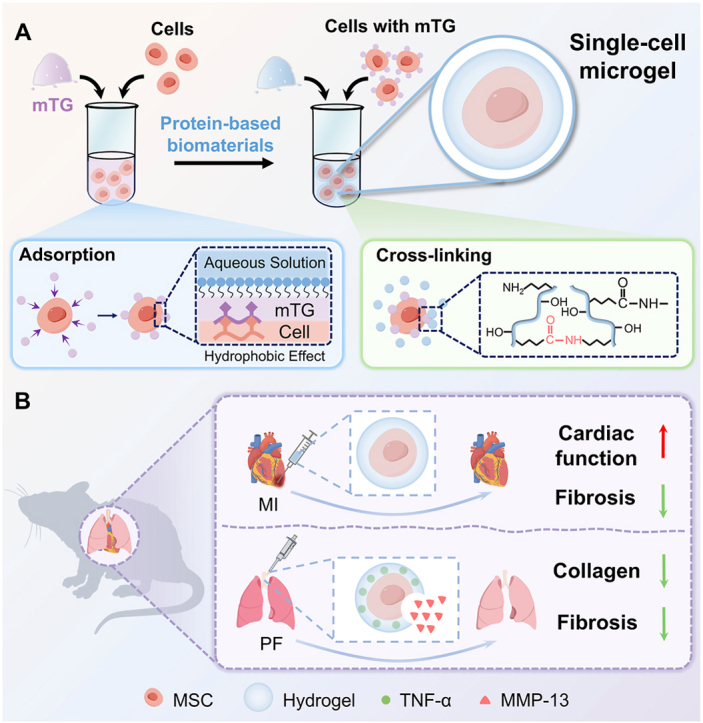

## 1 Introduction

Therapeutic delivery of drugs and small molecules to target tissues via various administration routes is a well-established paradigm for treating diverse pathologies. However, chronic conditions such as cancer and fibrosis demand sustained, long-term drug release for optimal therapeutic outcomes. Cell-based therapies have emerged as a transformative approach [[1], [2], [3]], leveraging their innate capacity for continuous molecular secretion and functional integration with host tissues-exemplified by clinical mainstays including blood transfusion and bone marrow transplantation. Notably, regulatory-approved modalities like stem cell therapy and CAR-T therapy have demonstrated unprecedented efficacy against treatment-refractory diseases [[4], [5], [6]]. Among cellular therapeutics, stem cells remain clinically preeminent due to their pluripotency and differentiation capacity, with wide applications in cardiovascular [7,8], respiratory [9,10], neurological [[11], [12], [13]], and musculoskeletal disorders [14]. Their therapeutic efficacy hinges on sustained functional integrity in vivo. However, the pathological microenvironment at the injury site impedes efficient delivery and viability of directly injected cells, resulting in markedly limited cell retention and survival. Clinical data demonstrate that even upon administration of up to 2 × 10^8^ stem cells, fewer than 5 % remain detectable in the tissue within hours after transplantation [15,16]. This poor viability leads to massive cell wastage and compromises clinical therapeutic outcomes.

Cell encapsulation in hydrogels has a long history in cell therapy. The clinical translation of cell encapsulation technology primarily addresses the critical challenge of donor shortage by enabling allogeneic transplantation. This innovative approach utilizes immunoisolation mechanisms to simultaneously shield transplanted cells from host immune rejection while ensuring sustained therapeutic molecule delivery, thereby offering a novel clinical paradigm for treating various refractory diseases. However, conventional three-dimensional (3D) bulk hydrogels are constrained by their nanoscale molecular networks, which significantly restrict nutrient diffusion, cellular viability, and bidirectional cellular migration-thereby compromising regenerative efficacy [17]. Microgels, typically defined as micron-scale (1–1000 μm) granular hydrogels, offer distinct advantages over bulk hydrogels for cell encapsulation in therapeutic applications [18,19]. Their spherical architecture maximizes the surface area-to-volume ratio, optimizing oxygen and nutrient exchange critical for cell survival. Clinically, this design facilitates minimally invasive cell delivery through standard injection protocols. The optimized spherical architecture minimizes foreign body responses while providing mechanical protection against shear stresses during administration. While microgel encapsulation of cell clusters addresses several limitations of conventional bulk encapsulation, its application remains constrained in spatially restricted anatomical sites (e.g., endobronchial, intravascular, or hippocampal delivery) where minimally invasive procedures demand precise size control. This creates a critical clinical challenge: maintaining functional efficacy while achieving the necessary size reduction of cell-loaded microgels.

Downscaling microgels to single-cell dimensions enables precise microenvironment mimicry and enhanced in vivo delivery, while providing exact control over encapsulation volume and ligand spatial distribution per cell. From a tissue engineering perspective, single-cell microgels (SCMs) of 50 μm diameter can achieve physiologically relevant cell densities of approximately 10^6^ cells/cm^3^ upon assembly [20]. SCMs, defined as hydrogel microgels encapsulating individual cells, offer a versatile strategy to effectively address these challenges [21,22]. Compared to conventional encapsulation carriers, microgels featuring thin hydrogel layers facilitate the transport of cell signaling and nutrient molecules, thereby enabling the regulation of cellular survival, proliferation, and migration. What's more, cell density represents another key determinant of tissue repair. Typically, high-density cell populations facilitate cell growth and extracellular matrix remodeling [23,24]. SCMs combine the advantages of both individual cell and microgel technology, enabling enhanced therapeutic cell payload delivery in confined injury sites while protecting the cells. However, scaling up the production of SCMs remains a critical translational bottleneck. Microfluidic strategies as a principal approach for SCMs fabrication generate droplets through oil-phase shearing [25,26]. However, this approach inherently suffers from Poisson distribution constraints, where low cell concentrations yield excessive empty microgels, while high concentrations lead to polydisperse cellular loading per microgel [27]. Importantly, cell viability is significantly impaired using oil and surfactants during fabrication, as well as the oil removal steps after preparation. Most recently, chemical modification, anchoring exogenous moieties to cell surfaces via covalent reactions, has also been widely adopted for SCMs fabrication due to its relatively high single-cell encapsulation efficiency [28,29]. It also faces problems with limited material selectivity, the cytotoxicity caused by harsh chemical conditions, and the interference with cell signaling pathways. Consequently, gentle fabrication strategies that preserve high single-cell encapsulation efficiency are vital for enhancing cell viability and therapeutic effect.

**Fig. 1:**
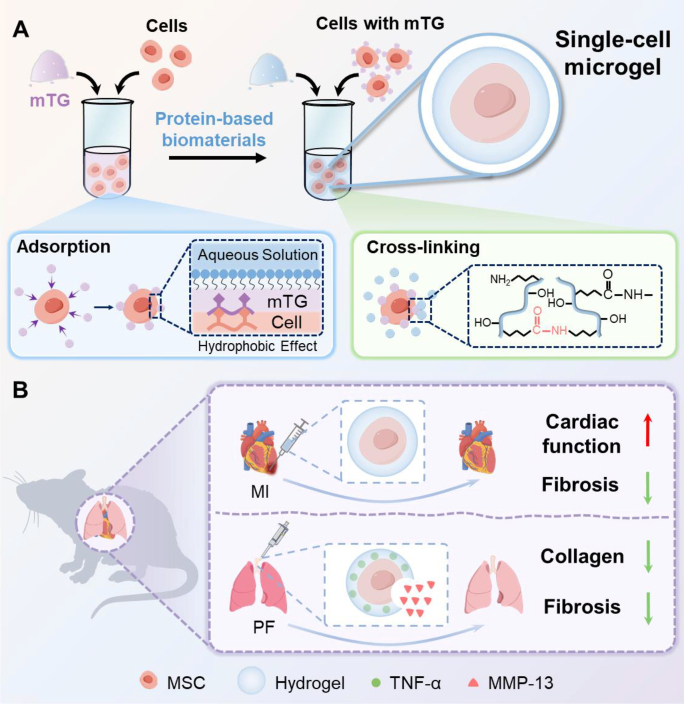
**Schematic for preparing SCMs via the bioenzymatic strategy. (A)** Cells were incubated in mTG solution to permit bioenzymatic adsorption, subsequently pelleted and resuspended in the solution of protein-based biomaterials for single-cell encapsulation. During this process, mTG catalyzes the cross-linking of protein molecules from outside the cell at 37 °C to form a gel on the cell surface. **(B)** SCMs for the treatment of myocardial infarction (MI) and pulmonary fibrosis (PF).

In this study, a simple and bioenzymatic strategy, based on microbial transglutaminase adsorption (“bioenzyme-mediated crosslinking”), is established for the fabrication of SCMs with high single-cell encapsulating efficiency and preserved cellular viability (Fig. 1). The method demonstrates broad compatibility with diverse cell types and hydrogel materials. To evaluate its therapeutic potential, we validated the efficacy of SCMs in disease models-MI (acute ischemic damage) and PF (chronic fibrotic remodeling, demonstrating significant tissue repair and regenerative outcomes. Notably, cell encapsulation markedly enhanced cell retention and survival rates within a pathological environment, indicating robust cytoprotection conferred by the hydrogel layer. Furthermore, SCMs effectively restored cardiac function and ameliorated PF, underscoring the clinical application promise of this approach for cell-based therapies.

## 2 Results

### 2.1 Engineering of bioenzymatic strategy and characterization of SCMs

**Fig. 2:**
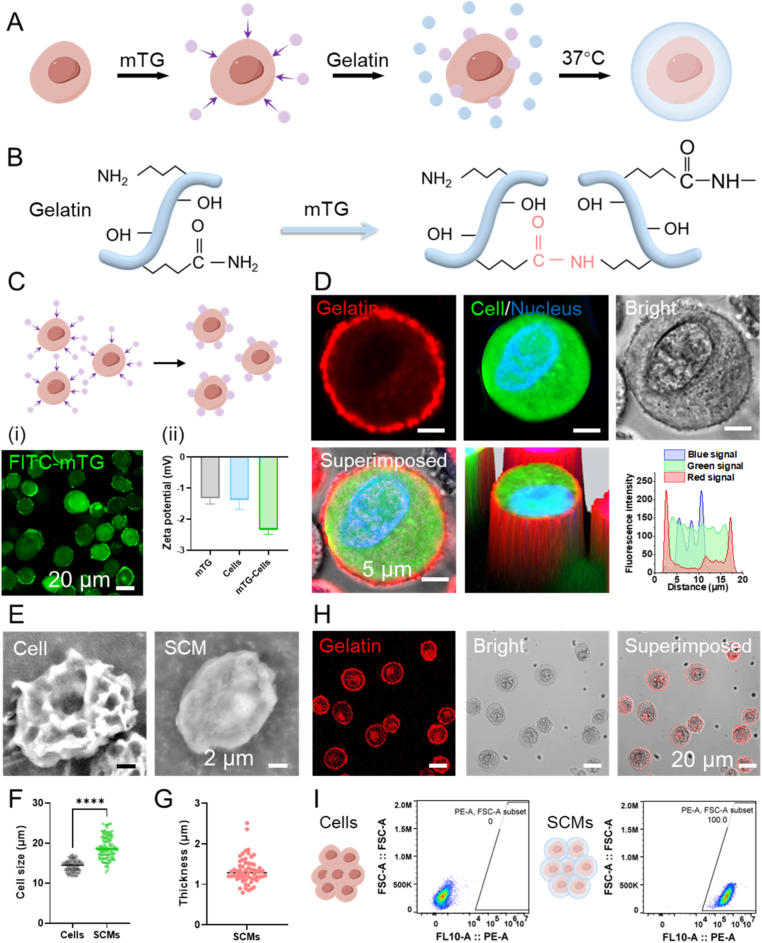
Characterization of **SCMs. (A)** Fabrication workflow of SCMs using gelatin. **(B)** Transamidation reaction between mTG and gelatin. **(C)** CLSM images and the zeta potential of the cells adsorbed with mTG (mTG-Cells). **(D)** CLSM images of SCMs (red: gelatin gel labeled with red fluorescent nanoparticles, green: cell stained by Calcein-AM, blue: cell nucleus stained by Hoechst 33342). **(E)** SEM images of the uncoated cell and SCM. **(F)** Size statistics of uncoated cells and SCMs, (n = 60). **(G)** The thickness of the hydrogel layer of MSC SCMs. A total of 20 cells were counted, and each cell was measured twice. **(H)** The fluorescence image and bright-field image of the SCMs. **(I)** Proportion of SCMs. The significant difference is determined by two-tailed unpaired t-tests. All data are means ± SD. ∗∗∗∗ *P* < 0.0001.

To achieve generalized SCMs fabrication and enhance the efficacy of cell-based therapies, we developed a bioenzymatic strategy based on microbial transglutaminase (mTG)-mediated crosslinking and physical adsorption principles (Fig. 2A and B). mTG is an FDA-approved enzyme with well-established biocompatibility [30]. The cell viability remained above 95 % (96.87 ± 1.64 %, Fig. S1A) in a 2 % mTG solution. During incubation, active mTG molecules spontaneously self-assemble onto cell surfaces via thermally driven diffusion and hydrophobic effects until saturation. FITC-mTG was employed to verify the successful adsorption of mTG molecules onto the cell surface (Fig. 2C i). To verify the adsorption mechanism of mTG on the cell surface, we first measured the zeta potential of cells before and after incubation with mTG. A decrease in zeta potential was observed, indicating that the adsorbed mTG molecules retained a net negative charge on the cell surface (Fig. 2C ii). To further explore this adsorption behavior, bovine serum albumin (BSA), a well-established model protein for protein-surface interaction studies, was introduced for comparison [31,32]. Similarly, incubation with BSA also resulted in a decrease in zeta potential, confirming the adsorption of negatively charged proteins onto the cell membrane (Fig. S1B). Given that BSA adsorption is largely governed by hydrophobic interactions, the observed mTG adsorption onto the cell surface is likely attributable to analogous nonspecific hydrophobic interactions. In addition, approximately 0.046 U of mTG could be adsorbed onto 1 × 10^6^ mesenchymal stem cells, confirming the enzyme-loading capacity of the cell surface (Fig. S1C). Subsequently, cells with mTG are introduced into hydrogel precursors (e.g., gelatin, collagen) capable of undergoing mTG-catalyzed crosslinking. Enzymes are confined to the cellular structure inducing localized hydrogel crosslinking at the cell surface to form a thin hydrogel layer around individual cell.

Among candidate biomaterials for cell encapsulation, gelatin serves as a representative model system. Gelatin, a low-cost, biodegradable, and biocompatible polymer derived from collagen hydrolysis, is an attractive biomaterial for cell-based therapies due to its native RGD (Arg-Gly-Asp) motifs that promote cell adhesion and migration [33]. To engineer cell-protective microenvironments, we exploited mTG to catalyze covalent ε-(γ-glutamyl)lysine cross-links between glutamine and lysine residues in gelatin at 37 °C. We preliminarily optimized the concentrations of gelatin and mTG and found that gelatin ≥10 % and mTG ≥1 % enabled efficient and uniform single-cell encapsulation (Fig. S2). Single-cell encapsulation was confirmed through triple fluorescence labeling with Hoechst 33342 (nucleus), Calcein-AM (viable cell), and red-fluorescent nanoparticles (hydrogel matrix). As shown in Fig. 2D, the SCMs were successfully generated by the bioenzymatic strategy. The SCMs revealed a smoother surface than uncoated cells (Fig. 2E and Fig. S3). In addition, the size of SCMs was significantly increased, with the thickness in hydrogel layer of 1.34 ± 0.32 μm (Fig. 2F and G). The size of the cells also clearly indicates that cells still maintained a single-cell status during gelation without aggregation. To assess single-cell encapsulation efficiency, uncoated cells and SCMs coated with a red fluorescent nanoparticle -labeled gelatin were quantified by Confocal laser scanning microscopy (CLSM) and flow cytometry. Critically, this bioenzymatic strategy overcomes the encapsulation efficiency bottleneck in conventional methods, which achieves 100 % single-cell encapsulation efficiency. Moreover, it greatly enhanced microgel production throughput, addressing critical manufacturing challenges in cell therapy commercialization (Fig. 2H–I and Fig. S4).

### 2.2 Versatile of the bioenzymatic strategy

**Fig. 3:**
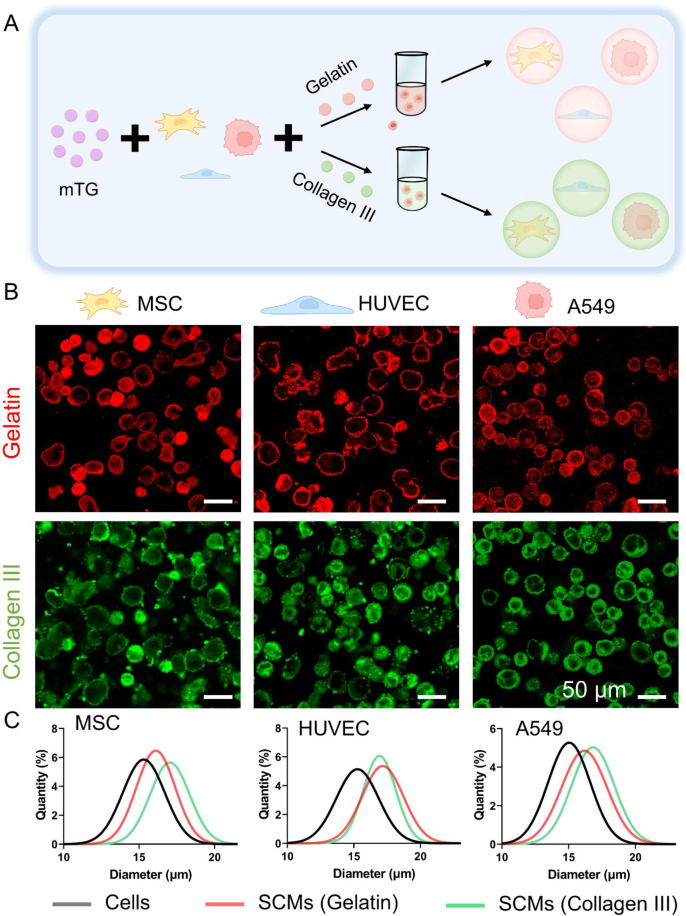
Characterization of SCMs encapsulated by gelatin and collage**n III. (A)** Schematic of SCMs fabricated with different materials. **(B)** Representative CLSM images of MSCs, HUVECs, and A549 encapsulated in gelatin and collagen hydrogels. **(C)** Size distribution of cells and SCMs. Gray, red and blue lines show distributions of cells, cells coated by gelatin, and cells coated by collagen III, (n = 20).

Cell lineage specificity is pivotal for cell therapy outcomes, governing therapeutic mechanisms, functional potency, target engagement, and host compatibility. For example, stem cells, owing to their multipotent differentiation capacity, are well-suited for regenerative medicine and tissue engineering; endothelial cells, leveraging their pro-angiogenic properties, can be utilized to construct vascularized tissue models or promote the repair of ischemic tissues; tumor cell lines provide essential foundations for establishing 3D tumor models in vitro and screening anticancer drugs. Therefore, the versatile of the SCMs fabrication strategy is pivotal for the efficacy of cell therapies and the accuracy of disease modeling. The core mechanism of the bioenzymatic strategy relies on physical adsorption-driven, non-specific cell encapsulation, circumventing the need for cell-surface modifications. To validate this generality, three distinct cell types were encapsulated (mesenchymal stem cells (MSCs), human umbilical vein endothelial cells (HUVECs), and A549 carcinoma cells). As shown in Fig. 3A–B and Fig. S5, this approach enables robust, high-throughput production of SCMs with different types of cells, in which all encapsulation efficiencies were >99 % regardless of cell type. Furthermore, within biomedical engineering, the selection of material is of pivotal importance, as it governs biocompatibility, functional integration, long-term stability, and ultimately, clinical application potential. For instance, gelatin, offering advantages over alginate due to its Arg-Gly-Asp (RGD) motifs, significantly enhances cell adhesion, spreading, and subsequent functional expression.

While traditional methods rely on electrostatic adsorption—restricted to rare cationic biomaterials like chitosan—our mTG-based approach enables different encapsulation across proteinaceous biomaterials, irrespective of charge. mTG catalyzes acyl transfer reactions, enabling the formation of crosslinks between protein-based biomaterials, offering features including biosafety, high substrate specificity, and mild reaction conditions. Consequently, this strategy enables cell encapsulation within any biomaterial susceptible to mTG-mediated crosslinking, eliminating material constraints in SCMs production. To demonstrate this universal applicability, we achieved equivalent single-cell encapsulation efficacy (100 %) using type III collagen (collagen III) as the gelling material. The robust production of SCMs demonstrates the potential generality of the bioenzymatic strategy, independent of cell type or material. Furthermore, the size before and after cell encapsulation revealed normally distributed (Fig. 3C). A rightward shift in size distribution was observed across each group relative to uncoated cells in varying degrees, these disparities are potentially attributable to differences in both material composition and cell type.

### 2.3 Cell behaviors of SCMs in vitro

**Fig. 4:**
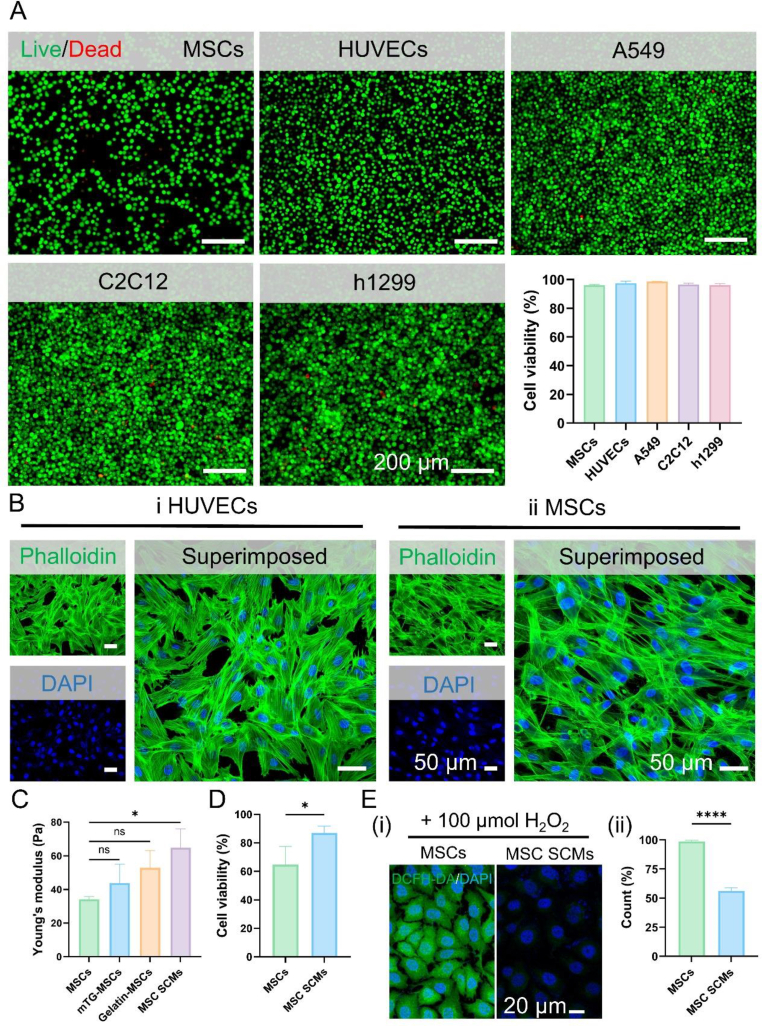
Cell behaviors of SCMs in **vitro. (A)** Fluorescence microscopy images and cell viability of the SCMs (the cells were stained with Calcein-AM/PI), (n = 3). **(B)** CLSM images of the HUVEC SCMs and MSC SCMs after 1 day of culture stained for F-actin (green) and nuclei (blue) with phalloidin and DAPI. **(C)** The mechanical properties of uncoated MSCs, mTG-MSCs, Gelatin-MSCs, and MSC SCMs were evaluated and compared by calculating the Young's modulus, (n = 3). **(D)** The viability of uncoated MSCs and MSC SCMs after being exposed to identical shear forces, (n = 4). **(E)** Fluorescence microscopy images and quantitative analysis of uncoated MSCs and MSC SCMs after treatment with H_2_O_2_ stained with DCFH-DA. The significant difference is determined by two-tailed unpaired t-tests and one-way analysis of variance (ANOVA) followed by Tukey's multiple comparison test. All data are means ± SD. ∗*P* < 0.05, ∗∗∗∗ *P* < 0.0001; ns, not significant.

To assess the impact of the bioenzymatic strategy on cell viability and function, cells with diverse functionalities were encapsulated within gelatin hydrogels. All encapsulated cell types (MSCs, HUVECs, A549, C2C12, and h1299) maintained >95 % viability at 24 h post-encapsulation (Calcein-AM/PI assay; respectively, 96.01 ± 0.60, 97.44 ± 1.43, 98.57 ± 0.17, 96.63 ± 0.78, 96.09 ± 1.05 %), validating the excellent biocompatibility of mTG-mediated crosslinking under standardized conditions (37 °C, pH 7.4, Fig. 4A). This strategy circumvents reliance on cell-surface-specific recognition or chemical modification, thereby minimizing interference with nature cell properties during encapsulation. To further validate this hypothesis, cells were co-stained with phalloidin and DAPI following 3 days of culture (Fig. 4B). The gentle encapsulation process preserved cytoskeletal integrity and cell-cell connections in both MSCs and HUVECs. These results demonstrate the excellent biocompatibility of the surfactant- and oil-free bioenzymatic approach, effectively preserving cell viability and functionality for therapeutic applications.

The fragility of cells often results in low survival rates after delivery, significantly compromising the efficacy of cell-based therapies. Consequently, we investigated the protective capacity for cells of the hydrogel layer via mechanical characterization. MSCs were encapsulated within a 10 % gelatin hydrogel using the bioenzymatic strategy. A progressive increase in stress with greater compressive displacement (Fig. S6). As shown in Fig. 4C, MSC SCMs exhibited a significantly increased Young's modulus compared with uncoated MSCs, whereas only-enzyme (mTG-MSCs) and only-gel (Gelatin-MSCs) controls without significant differences, confirming that the mechanical reinforcement arises from the crosslinked hydrogel layer. To further evaluate protection under flow conditions, a fluid shear stress assay was performed. Following exposure to identical shear forces, uncoated MSCs and MSC SCMs were stained for viability assessment. Results confirmed a significant reduction in survival within the control group (64.89 ± 12.63 %), while encapsulated cells maintained higher viability (87.01 ± 4.89 %), demonstrating the hydrogel's protective function (Fig. 4D).

Beyond providing mechanical protection, we hypothesized that the microgel encapsulation strategy would confer robust resistance to oxidative stress, demonstrating its exceptional adaptability to extreme pathological microenvironments. Oxidative stress represents a common pathological underpinning of numerous diseases, including cardiovascular and neurodegenerative disorders. To evaluate the antioxidative protective capacity of SCMs, we employed hydrogen peroxide (H_2_O_2_)-induced oxidative stress models. Following 24 h incubation with 100 μM H_2_O_2_, intracellular reactive oxygen species (ROS) levels were assessed using DCFH-DA staining. As depicted in Fig. 4E, H_2_O_2_ treatment significantly elevated cell ROS content due to oxidative damage. In contrast, MSC SCMs markedly reduced ROS levels relative to uncoated controls, demonstrating effective protection against oxidative stress. Notably, the crosslinking of the gelatin hydrogel layer is mediated by stable covalent bonds, rendering the hydrogel network structurally robust under oxidative stress conditions. The protective effect of the gelatin hydrogel against oxidative stress may arise from two complementary mechanisms. First, gelatin contains amino acid residues with mild reducing capability, endowing the hydrogel with intrinsic but modest antioxidative activity. Second, the hydrogel layer serves as a physical diffusion barrier that retards the direct contact of H_2_O_2_ with the cell membrane. A higher concentration of gelatin may result in the formation of a denser network structure, thereby enhancing the antioxidant protection ability (Fig. S7). For long-term oxidative environments, this platform offers the flexibility to incorporate exogenous antioxidant agents within the hydrogel matrix, thereby further enhancing resistance to oxidative stress when required.

Collectively, these findings demonstrate that thin-layer hydrogel encapsulation through our bioenzymatic approach provides multi-faceted cytoprotection, simultaneously enhancing mechanical robustness, mitigating shear-induced damage during delivery, and preserving cell viability under oxidative stress, thereby addressing critical bottlenecks in therapeutic cell applications. We further investigated the stability and degradability of the hydrogel layer. Real-time live-cell imaging revealed that SCMs were able to gradually breach the gelatin layer and exhibit a well-spread morphology within 48 h (Fig. S8A). To assess in vivo degradation behavior, SCMs were subcutaneously injected and the degradation profile of the gelatin layer was monitored. FITC-labeled gelatin exhibited gradual degradation over time and was nearly completely degraded within 36 h, confirming the favorable biodegradability of the gelatin hydrogel layer in vivo (Fig. S8B).

### 2.4 Transcriptomic profiling of the effects of gelatin encapsulation on MSCs

To further assess the functional impact of gelatin encapsulation, we conducted transcriptomic sequencing on cells after 72 h of encapsulation. We analyzed two groups: uncoated MSCs (MSCs) and gelatin-encapsulated MSCs (MSCs SCMs). Differential expression analysis identified 194 upregulated and 380 downregulated genes in encapsulated cells relative to controls (Fig. S9A–B). Gene Ontology (GO) enrichment of these differentially expressed genes (DEGs) indicated upregulated functions in cell proliferation and repair, spanning the biological processes “cell cycle”, “cell division”, and “DNA repair”; the cellular components “centromere region”, “kinetochore”, and “chromosome”; and the molecular functions “microtubule motor activity” and “nucleotide binding” (Fig. S9C). Functions related to the extracellular matrix and intercellular communication were downregulated, including the biological processes “extracellular matrix organization”, “cell adhesion”, and “potassium ion transport”; the cellular components “extracellular region”, “extracellular matrix”, and “extracellular space”; and the molecular functions “calcium ion binding”, “extracellular matrix structural constituent”, and “potassium channel activity” (Fig. S9D). Kyoto Encyclopedia of Genes and Genomes (KEGG) pathway analysis of the top 10 enriched pathways revealed upregulated pathways associated with the cell cycle and inflammatory regulation, such as “cell cycle”, “oocyte meiosis”, “IL-17 signaling pathway”, and “TNF signaling pathway” (Fig. S9E). In contrast, pathways involved in extracellular matrix maintenance and communication, including “ECM-receptor interaction”, “calcium signaling pathway”, and “focal adhesion” were downregulated (Fig. S9F). These findings suggested the encapsulated cells enter a proliferative state at this stage, establishing a foundation for subsequent functional activities.

To evaluate the effect of encapsulation on cell identity, we examined genes encoding characteristic MSC surface markers. Encapsulation left Nt5e expression unchanged but increased the expression of Ly6a, Itgb1, Cd44, and Thy1 (Fig. S10A). The stemness maintenance gene Nanog was also unaffected (Fig. S10B). These findings indicate that encapsulation does not impair, and may even aid in, maintaining the MSC phenotype. Analysis of lineage-specific differentiation genes—covering osteogenic (Runx2, Sp7), chondrogenic (Sox9, Col2a1), and adipogenic (Pparg, Cebpa) markers—showed no significant changes in Sp7, Sox9, or Pparg, whereas Runx2, Col2a1, and Cebpa were upregulated (Fig. S10C–E), implying that encapsulation does not suppress and could potentially enhance MSC differentiation capacity. Among immune regulatory genes, Cxcl9 expression remained stable, while Cxcl10 was elevated (Fig. S10F), suggesting encapsulation does not obstruct and may instead foster immunomodulatory functions. For genes encoding secreted proteins, Hgf was downregulated, whereas Vegfa and Vegfc were upregulated (Fig. S10G), indicating that encapsulation may steer cells toward pro-angiogenic and pro-lymphangiogenic activities.

### 2.5 SCM-based therapeutic applications in acute and chronic disease models

Cell therapy stands as a revolutionary therapy of regenerative medicine, holding immense potential for treating refractory pathologies including degenerative disorders, tissue injuries, immune dysregulation, and malignancies [34,35]. However, its broad clinical application remains constrained by a key therapeutic bottleneck—inefficient cellular delivery and poor post-transplant survival. To demonstrate the significance of our SCM platform in overcoming this fundamental limitation, we verified its efficacy in cardiopulmonary disease models (MI and PF) representing cardinal clinical challenges. In treating MI, the prevalent ischemic/hypoxic microenvironment drastically reduces cell survival [36,37]. Furthermore, conventional delivery methods, namely delivery cell carriers via intravenous or intramyocardial injection, are hampered by limitations including the risk of vascular obstruction and the restricted capacity of the cardiac chamber. A substantial proportion of transplanted cells perish rapidly after administration, severely compromising delivery efficiency and overall therapeutic efficacy [38,39]. In the PF, the therapeutic efficacy of MSCs is intrinsically linked to inflammatory microenvironmental cues—their paracrine activity (mediated by secreted factors and exosomes diminishes as inflammation resolves during disease progression [40]. This inflammation-dependence fundamentally limits sustained therapeutic impact, particularly in late-stage fibrotic remodeling where collagen degradation becomes critical. We hypothesize that our SCM platform addresses two fundamental limitations of MSC therapy in PF: (i) rapid cell dispersion from target sites, and (ii) inflammation-dependent paracrine activity. The successful fabrication of SCMs offers a promising approach to address these challenges. The subsequent studies rigorously assess the therapeutic potential of SCM-based therapy for both MI and PF.

## 3 SCM-based therapy promotes cardiac repair after MI

### 3.1 Single-cell microencapsulation enhances MSC retention in MI

**Fig. 5:**
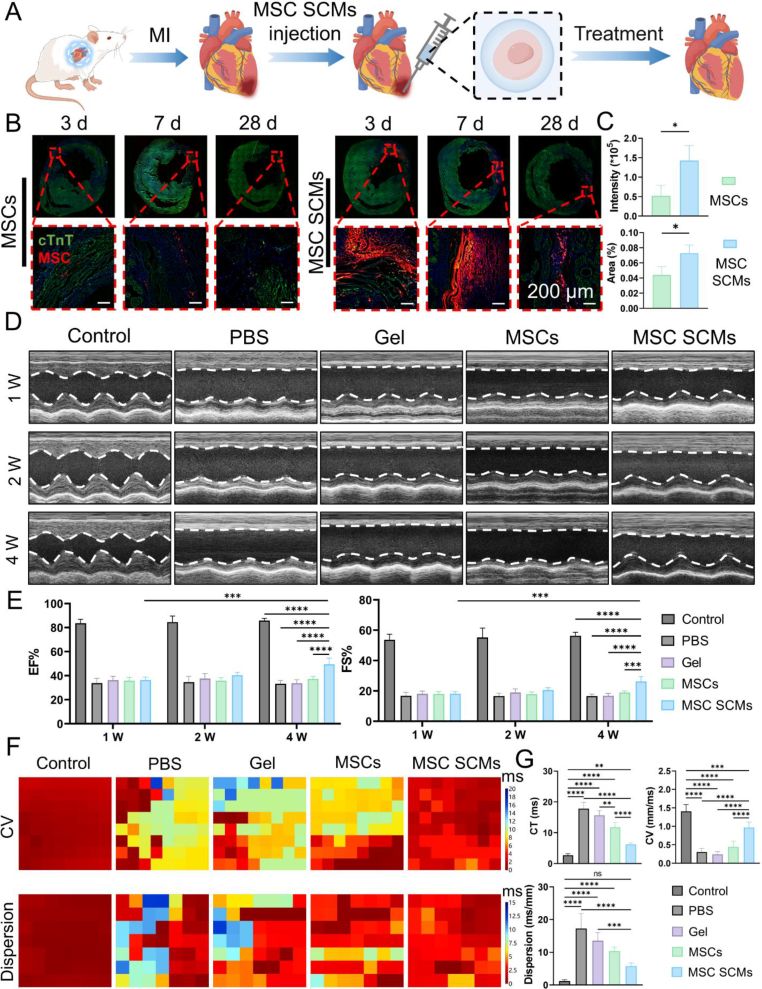
Enhancing MSC retention and improving cardiac function by MSC SCMs post-MI in **vivo. (A)** Schematic of SCMs treatment of MI. **(B)** Representative images of MSCs tracked by CM-DID at 3 d, 7 d and 28 d post-MI. **(C)** Quantitative analysis of CM-DiD fluorescence intensity and distribution area at 7 d post-MI. **(D)** M‐mode echocardiographic images of rats at 1-, 2- and 4-weeks post-MI. **(E)** Statistical analysis of LVEF and LVFS post-MI, (n = 5). **(F)** Representative electrical activation and dispersion maps of left ventricular at 4 weeks after MI (the earliest activations or dispersions are shown in red, while the latest appear in blue, with numerical values indicating the timing of activation or dispersion in milliseconds (ms)). **(G)** Statistical analysis of conduction time (CT), conduction velocity (CV), and conduction dispersion at 4 weeks post‐MI with/without treatment, (n = 5). The significant difference is determined by ANOVA followed by Tukey's multiple comparison test. All data are means ± SD. ∗*P* < 0.05; ∗∗*P* < 0.01; ∗∗∗*P* < 0.001; ∗∗∗∗*P* < 0.0001; ns, not significant.

We applied SCMs to the MI model (Fig. 5A). Our study hypothesized that single-cell hydrogel encapsulation would significantly improve MSC retention in target tissues. To test this hypothesis, we conducted systematic in vivo tracking of CM-DiD-labeled MSCs in a rat MI model, comparing microencapsulated (MSC SCMs) versus unencapsulated cell formulations. Quantitative retention kinetics were established through longitudinal fluorescence imaging at 3-, 7-, and 28-days post-transplantation, with particular focus on the critical first-week window for cell engraftment. While both groups exhibited expected temporal cell loss patterns, MSC SCMs treatment yielded significantly higher cellular retention than the MSCs group throughout the observation period (Fig. 5B). Quantitative analysis at day 7 revealed greater CM-DiD fluorescence intensity and distribution area in the MSC SCMs-treated hearts (increasing by 175.27 % and 63.37 % respectively compared to the MSCs group, Fig. 5C). Lamin A + C (Human Nuclear Envelope Marker) staining of myocardial tissue at day 7 after injection further demonstrated that the proportion of Lamin A + C-positive cells was significantly higher in the MSC SCMs group than in the MSCs group (Fig. S11). This result aligns with the trends observed for CM-DiD fluorescence intensity and distribution area. Collectively, these findings confirm that gel encapsulation enhances the retention of MSCs. CM-DiD tracking demonstrated that at 7 days post-transplantation, MSCs primarily localized to cardiac tissue in the MSC SCMs group, with limited extra-cardiac distribution (Fig. S12A). In contrast, the MSCs group showed substantial pulmonary accumulation (Fig. S12B), suggesting systemic dispersion despite the absence of clinical complications. We speculate that gel encapsulation alters the cellular size, morphology, and surface protein expression, potentially impeding the entry of MSC SCMs into the permeable vasculature and their subsequent migration to the lungs. This may lead to their retention within the myocardial tissue. The significantly enhanced retention and targeted localization properties of MSC SCMs establish a robust foundation for improved therapeutic outcomes in MI treatment, potentially addressing a major clinical challenge in cardiac cell therapy by overcoming the critical limitation of poor cell retention in current regenerative approaches.

### 3.2 MSC-loaded SCMs restore cardiac function in MI models

To investigate the therapeutic potential of MSC SCMs in MI, we intramyocardially injected MSC SCMs post-MI and monitored cardiac function using echocardiography and cardiac electrical propagation measurements. Following the experimental timeline, echocardiographic evaluations were conducted at 1-, 2- and 4-weeks post-MI. Echocardiography demonstrated comparable and significant reductions in left ventricular function across all groups first week post-MI (Fig. 5D). Quantitative analysis revealed no intergroup differences in LVEF or LVFS during this phase (Fig. 5E). By the second week, MSC SCMs-treated rats exhibited early signs of functional recovery, which became more pronounced by week 4: LVEF rose from 36.32 ± 2.47 % to 49.47 ± 5.09 %, while LVFS increased from 18.21 ± 1.45 % to 26.26 ± 3.26 %. The MSC SCMs group outperformed all other treatment groups, demonstrating its superior potential for functional restoration post-MI.

Four weeks post-MI, ventricular conduction properties were analyzed. Compared to wild-type controls (Control group), all MI groups exhibited prolonged conduction time (CT), slower conduction velocity (CV), and increased dispersion (Fig. 5F and G). While MSCs and MSC SCMs treatments partially reversed these abnormalities, with MSC SCMs show the better recovery: CT decreased to 6.238 ± 0.4794 ms, CV improved to 0.9649 ± 0.1511 mm/ms, and dispersion diminished to 5.752 ± 0.9821 ms/mm—values closer to those of control groups. In contrast, the gelatin microgel group (Gel group) showed no electrophysiological benefits, and the naked MSCs group (MSCs group) provided only modest improvements. These findings substantiate the therapeutic advantage of MSC SCMs in restoring post-MI ventricular conductivity.

### 3.3 MSC microgels preserve ventricular architecture post-MI

**Fig. 6:**
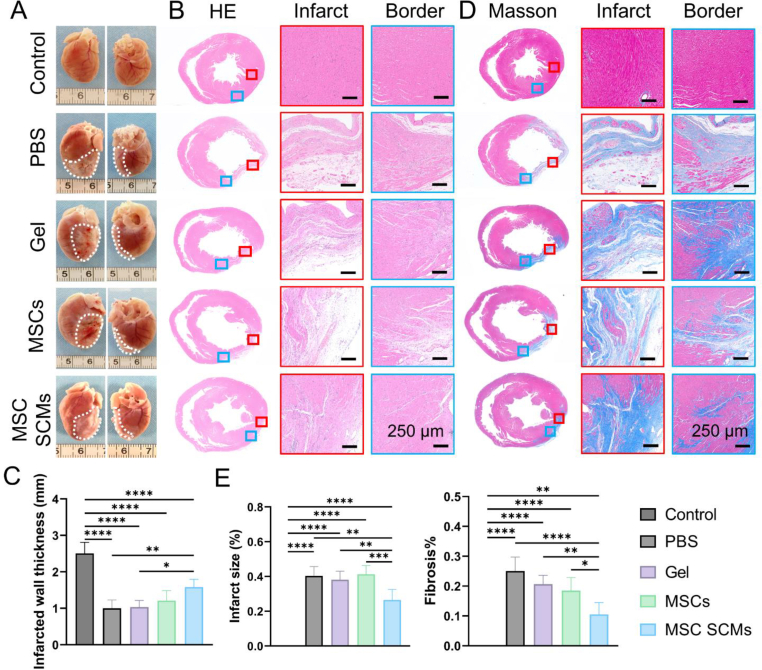
Maintaining cardiac morphology following MI via MSC SCMs in **vivo. (A)** Gross morphology of hearts harvested at 4 weeks post-MI. **(B)** Representative images of hematoxylin and eosin‐stained cross‐sections of the infarcted ventricular myocardium at 4 weeks post-MI. **(C)** Quantitative analysis of infarcted wall thickness across different groups, (n = 5). **(D)** Representative images of Masson's trichrome‐stained cross‐sections of the infarcted ventricular myocardium at 4 weeks post-MI. **(E)** Quantitative analysis of infarct size and fibrosis extent across different groups, (n = 5). The significant difference is determined by one-way ANOVA, followed by Tukey's test. All data are means ± SD.∗*P* < 0.05; ∗∗*P* < 0.01; ∗∗∗*P* < 0.001; ∗∗∗∗*P* < 0.0001; ns, not significant.

Hearts were harvested for evaluating tissue morphology four weeks post-MI. Gross examination revealed smaller infarct areas in MSC SCMs-treated hearts compared to other groups (Fig. 6A). Hematoxylin-eosin staining revealed more extensive tissue regeneration in the MSC SCMs group (Fig. 6B), with significantly increased ventricular wall thickness (Fig. 6C). Masson's trichrome staining indicated reduced left ventricular scar area and diminished fibroblast infiltration in MSC SCMs-treated hearts compared to other groups (Fig. 6D and E). These findings collectively indicate that MSC SCMs treatment mitigated fibrosis while preventing myocardial thinning post-MI. Histological assessment of liver, spleen, lungs, and kidneys 28 days post-treatment revealed no pathological changes (Fig. S13), confirming the biocompatibility of MSC SCMs transplantation for therapeutic applications. The combined data demonstrate that MSC SCMs transplantation improved cardiac function and preserved cardiac morphology post-MI without inducing systemic toxicity.

## 4 SCM-enhanced MSC therapy for PF via cytokine synergy

### 4.1 TNF-α potentiates MSC paracrine function via single-cell microencapsulation

PF is driven by ECM dysregulation, wherein hyperactivated fibroblasts pathologically overproduce and deposit collagen. Targeting this aberrant collagen accumulation represents a critical therapeutic challenge, as conventional anti-fibrotics fail to address established matrix pathology. Of note, MMP-13 is a specific collagen-degrading enzyme that can cleave collagen at specific sites within the triple helical domain. Therefore, given its unique capacity to degrade pathological collagen matrices, MMP-13 emerges as a primary regulator for PF resolution. Inflammatory microenvironment constitutes a critical element of stem cell paracrine signaling dynamics, particularly for MSCs. Research has shown that tumor necrosis factor-alpha (TNF-α) as one of the predominant inducers of MMP-13 secretion in MSCs, demonstrating significantly superior inducing effect over interferon-gamma (IFN-γ), interleukin-1 beta (IL-1β), and lipopolysaccharide (LPS) [41].

**Fig. 7:**
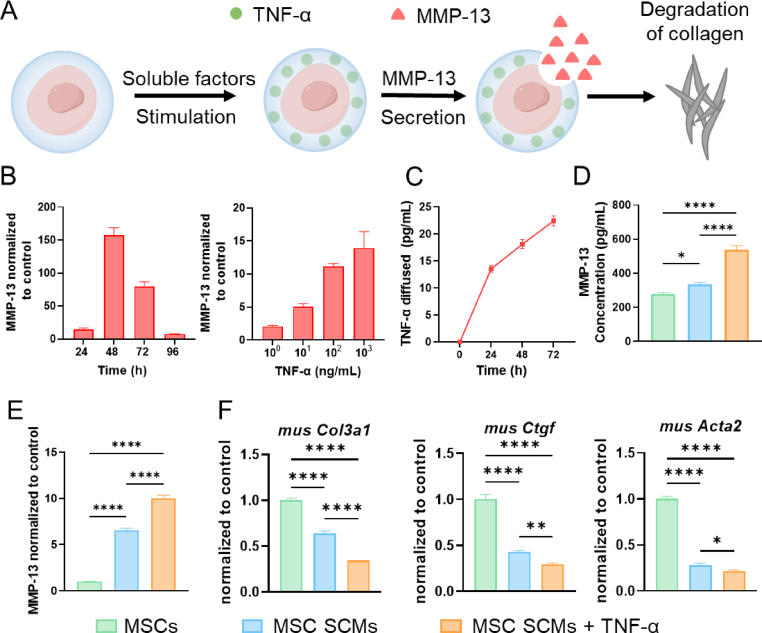
The induction effect of TNF-α on MSCs in **vitro. (A)** The TNF-α loaded in the SCMs promotes the paracrine effect of MSCs, augmenting the secretion of MMP-13 to drive collagenolytic degradation. **(B)** MMP-13 mRNA expression at gradient concentrations of 1–1000 ng/mL and at 1–4 days of culture, (n = 3). **(C)** The release behavior of TNF-α in MSC SCMs + TNF-α, (n = 3). **(D)** The secretion of MMP-13 protein, (n = 3). **(E)** MMP-13 mRNA expression of MSCs, MSC SCMs, and MSC SCMs + TNF-α, (n = 3). **(F)** Expression of profibrogenic gene mRNA after MSCs, MSC SCMs, and MSC SCMs + TNF-α groups of treatments in vitro, (n = 3). The significant difference is determined by one-way ANOVA, followed by Tukey's test. All data are means ± SD. ∗*P* < 0.05; ∗∗*P* < 0.01; ∗∗∗∗*P* < 0.0001.

Therefore, we examined the potential for MSCs to release MMP-13 via paracrine signaling following TNF-α induction (Fig. 7A). MMP-13 mRNA expression exhibited a concentration-dependent elevation with increasing concentrations of TNF-α. Notably, the cumulative increase in MMP-13 secretory output observed at 100 ng/mL TNF-α proved significantly more potent than that elicited by 1000 ng/mL (100 ng/mL increase rate: 121.29 %; 1000 ng/mL increase rate: 25.23 %). In addition, MMP-13 secretion peaked on day 2 after TNF-α induction (Fig. 7B). Based on this, leveraging TNF-α-mediated MSC potentiation and the loading capacity of MSC SCMs, we proposed a strategy of the combination of cells and cytokines to augment therapeutic outcomes. TNF-α release from the TNF-α-loaded MSC SCMs (MSC SCMs + TNF-α) was first evaluated by ELISA, which revealed a sustained and slow-release profile (Fig. 7C). To further assess the in vivo safety of this delivery strategy, MSC SCMs + TNF-α were administered to mice, and hematoxylin and eosin (H&E) staining performed three days post-injection demonstrated that TNF-α release did not elicit detectable local inflammatory responses (Fig. S14). Compared to both uncoated cells and MSC SCMs, MSC SCMs + TNF-α substantially increased the levels of MMP-13 gene expression and protein secretion (Fig. 7D and E). Moreover, MSC SCMs also exhibited markedly elevated MMP-13 secretion compared with uncoated cells. This enhancement is likely attributable to the 3D environment of the SCMs and the mechanical signal transduction (e.g., substrate stiffness), which enhances the paracrine activity of the stem cells. To preliminarily assess the anti-fibrotic capacity of SCMs, a fibroblast fibrotic model was established via TGF-β induction. A marked downregulation of profibrogenic gene expression following treatment with both MSC SCMs and MSC SCMs + TNF-α (Fig. 7F).

### 4.2 SCMs promote collagen degradation in PF

**Fig. 8:**
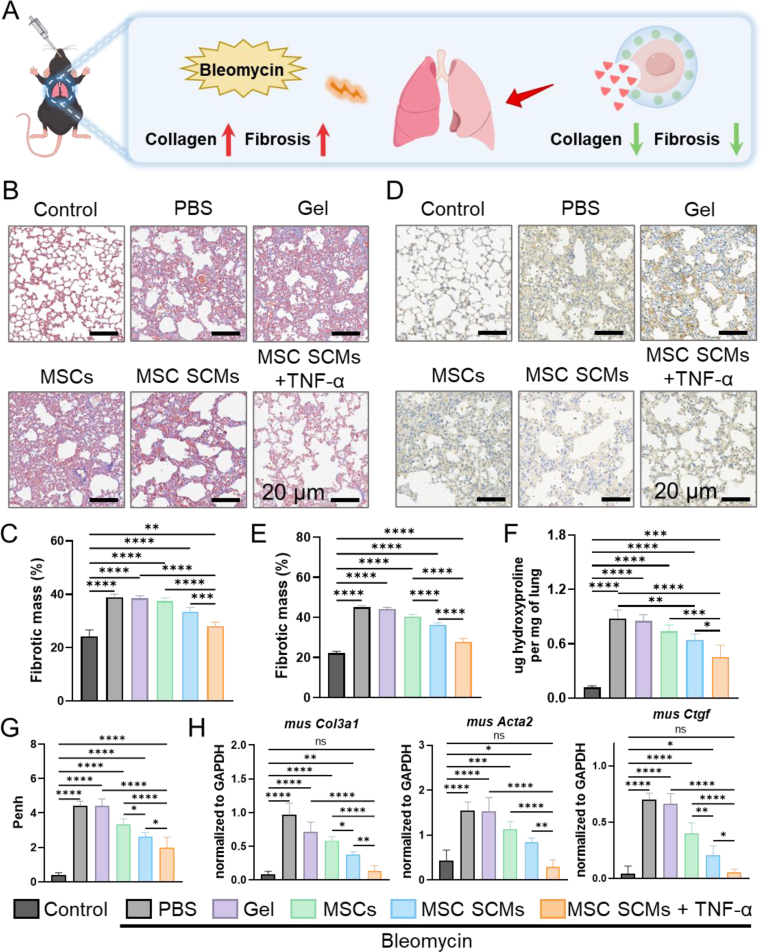
Promoting tissue remodeling of PF via **SCMs. (A)** Schematic of SCMs treatment of PF. **(B**–**C)** Representative images and percent total fibrotic mass from Masson's trichrome stain, (n = 5). **(D**–**E)** Representative images and percent total fibrotic mass from collagen stain, (n = 5). **(F)** Hydroxyproline levels of lung tissue, (n = 5). **(G)** Penh in mice, (n = 6). **(H)** Expression of profibrogenic gene mRNA in lung tissue, (n = 6). The significant difference is determined by one-way ANOVA, followed by Tukey's test. All data are means ± SD. ∗*P* < 0.05; ∗∗*P* < 0.01; ∗∗∗*P* < 0.001; ∗∗∗∗*P* < 0.0001; ns, not significant.

Having established the enhanced paracrine activity and anti-fibrotic potential of MSC-loaded SCMs in vitro, we next assessed therapeutic efficacy in vivo using a bleomycin-induced PF mouse model (Fig. 8A). Upon confirmation of bleomycin-induced pathology (as evidenced by sustained weight loss; Fig. S15), we administered 100,000 microgels without cells, uncoated MSCs, MSC SCMs, or TNF-α-primed MSC SCMs per 20 g body weight to further investigate the ability of SCMs to degrade collagen deposition. To assess collagen deposition, Masson's trichrome and collagen immunohistochemical staining were performed one week after delivery. Microgel carriers without cells provided no therapeutic benefit, confirming that the hydrogel material alone does not mitigate PF. In contrast, both MSC SCMs and MSC SCMs + TNF-α substantially reduced fibrotic lesion area compared to the PF model and uncoated MSC groups, restoring alveolar architecture approaching that of healthy controls (∼20 %, Fig. 8B–E). Hydroxyproline, a major constituent of collagen, served as a biochemical marker for total collagen content. Consistent with histopathological analyses, bleomycin challenge resulted in an increase in hydroxyproline, whereas MSC SCMs + TNF-α treatment significantly reduced, almost returning to the level of healthy controls (from 0.40 ± 0.09 to 0.19 ± 0.25 μg/mg, Fig. 8F). Concurrently, assessment of respiratory function parameters (Penh) revealed that MSC SCMs improved pulmonary function, with optimal enhancement observed in the MSC SCMs + TNF-α group (Fig. 8G). qRT-PCR analysis confirmed in vivo downregulation of myofibroblast biomarkers (*Col3a1, Acta2, Ctgf*), aligning with prior in vitro findings (Fig. 8H). In addition, major organs exhibited no detectable pathological alterations in the SCMs group compared to healthy controls, establishing the SCM platform as a secure delivery system for cell therapy (Fig. S16). These outcomes in vivo indicate that the anti-fibrotic efficacy of MSC SCMs against bleomycin-induced injury stems from their enhanced paracrine activity. This augmentation observably improves levels of the collagen-degrading enzyme MMP-13, facilitating tissue remodeling.

## 5 Discussion

Cell therapy has demonstrated significant potential in treating a spectrum of disorders, with emerging clinical applications in cardiovascular regeneration, pulmonary disease, neurodegenerative conditions, and musculoskeletal repair. However, its clinical translation remains hindered by poor cell retention, limited scalability, and dependency on complex manufacturing processes. Here, we present a bioenzymatic SCM platform that addresses these challenges through universal applicability, scalable production, and clinical-grade simplicity, offering a robust solution for next-generation cell therapies. This study introduces a versatile bioenzymatic strategy that enables the robust fabrication of SCMs, effectively overcoming the major limitations of conventional cell encapsulation approaches. By leveraging mTG-mediated physical adsorption and localized crosslinking, we achieved universal single-cell encapsulation across diverse cell types and protein-based biomaterials, without the use of cytotoxic surfactants, oils, or harsh chemical modifications. Compared with other stem cell engineering strategies, such as layer-by-layer (LBL) encapsulation [42], bio-orthogonal surface reactions [43], and micropatch attachment [44,45], our approach emphasizes simplicity, universality, and high efficiency. LBL and bio-orthogonal strategies often involve multistep processing or synthetic chemical modifications, while micropatch-based methods typically require sophisticated fabrication and are suited for asymmetric surface functionalization. In contrast, the mTG-mediated strategy enables rapid, uniform, and degradable single-cell encapsulation without requiring complex chemical modifications. Moreover, compared with microfluidics, this strategy eliminates the need for complex chips or instruments, offering advantages in operational simplicity and low cost. The resulting microgels, characterized by a thin hydrogel layer, preserve high cellular viability and cellular functionality, including cytoskeletal integrity and cell-cell communication, thereby addressing the critical bottleneck of cellular damage during fabrication encountered with microfluidic (microchannel) or covalent anchoring strategies. This thin hydrogel encapsulation demonstrated its strong cytoprotective effects. It markedly enhanced cell mechanical properties, as evidenced by increased Young's modulus, enabling encapsulated cells to withstand shear forces encountered during delivery. Critically, the microgels provided effective protection against the pathological microenvironments, such as oxidative stress. MSC SCMs demonstrated significantly reduced ROS accumulation under H_2_O_2_ challenge compared to unencapsulated controls, confirming the hydrogel layer's protective efficacy against oxidative stress.

These protective attributes and the loading capacity of the hydrogel layer were directly verified into superior therapeutic efficacy in models of MI (acute ischemic damage) and PF (chronic fibrotic remodeling). The SCM platform dramatically enhanced cell retention and persistence within the pathological microenvironment, addressing a fundamental weakness of conventional cell delivery and therapy. In MI, this improved retention directly translates to superior therapeutic outcomes: MSC SCMs administration markedly restored cardiac function post-MI, while electrophysiological mapping revealed near-normalization of conduction velocity and dispersion. Concurrently, MSC SCMs treatment preserved cardiac morphology, reducing infarct size. In PF, we further utilized the microgel platform's capabilities by integrating cytokines. The encapsulation within the gelatin hydrogel improved the paracrine activity of MSCs, likely due to enhanced mechanical signaling or a 3D conducive environment. MSC SCMs with TNF-α synergistically unleashed maximal MMP-13 secretion. This innovative combination strategy (MSC SCMs with TNF-α) exhibited remarkable anti-fibrotic outcomes, including significant collagen degradation, and restoration of lung architecture and function. This bioenzymatic single-cell microencapsulation strategy may also be applicable to broader stem cell engineering scenarios. Recent advances have demonstrated that biomaterials can enable in situ modulation of stem cell fate and function [46], as well as neural tissue regeneration through microenvironmental regulation [47]. While the thin pericellular hydrogel layer provided by SCMs offers a potential means to engineer stem cells for in situ cell therapy and neural tissue engineering.

mTG exhibits exceptional catalytic efficiency, stringent substrate specificity, and robust stability under physiological conditions. These attributes, combined with its capacity for site-selective protein crosslinking, position mTG as an ideal biocatalyst for cell surface engineering. The resulting hydrogel layer exhibits a nanoscale mesh structure that allows the diffusion and activity of exosomes, chemokines, ligands, and other signaling molecules while not completely obstructing their passage. Furthermore, the homing capacity of MSCs, primarily governed by chemokine-receptor interactions, is likely maintained, as the hydrogel does not hinder factor diffusion and degrades within 36–48 h. This degradable nature of the hydrogel layer ensures that the encapsulation protects the cells without altering the homing or secretory functions of MSCs.

Future screening of encapsulating materials will enable this bioenzymatic platform to substantially expand the fabrication scope and therapeutic applicability of SCMs, particularly for precision tissue regeneration and personalized cell therapies. While this study offers a highly generalizable platform for next-generation cell therapies, certain limitations warrant future exploration. Long-term monitoring of transplanted SCMs, potential immune responses, and the precise biodegradation kinetics of the hydrogel layer in different tissues require further investigation. Optimizing hydrogel composition, stiffness, and degradation profiles to further tune cell-matrix interactions and controlled release of active substances (such as cytokines, exosomes, and enzymes) could enhance therapeutic precision. Overall, this bioenzymatic single-cell microencapsulation strategy represents a crucial shift, offering a biocompatible, universal, scalable, simple, and cytoprotective platform to overcome the critical delivery and survival barriers in cell-based regenerative therapies.

## 6 Materials and methods

### 6.1 Animal ethics statement

Male C57BL/6 mice (6 weeks old) were supplied by GemPharmatech Co., Ltd. (12 Xuefu Road, Pukou District, Nanjing 210061, China) in accordance with guidelines from the Laboratory Animal Center of Guangzhou Medical University (Guangzhou, China). All experimental procedures were approved by the Institutional Animal Care and Use Committee of Guangzhou Medical University (Approval No. GY2024-315; Animal License No. SYXK 2025-0168). Male Sprague-Dawley rats (200–250 g) were obtained from Shanghai Jihui Experimental Animal Co., Ltd. All procedures complied with the guidelines of the Animal Care and Experiment Committee of the Shanghai Children’s Medical Center (Approval No. SCMC-LAWEC-2021-011).

### 6.2 Materials

Porcine skin gelatin (V900863-500G) and cold-water fish skin gelatin (G7041-500G) were purchased from Sigma-Aldrich (USA). Type III collagen was obtained from Hebei NACOL Biotechnology Co., Ltd; mTG (BC5582) was sourced from Hefei Bomei. The DCFH-DA probe (S0033S), Cell Live/Dead Staining Kit (C2015M), and Actin-Tracker reagent (C2203S) were provided by Beyotime Biotechnology (China). Red fluorescent nanoparticles (FH107OR) were acquired from Rigor Biotechnology (China). Bleomycin (S1214) was ordered from Selleck (USA). The Hydroxyproline Content Assay Kit (BC0255) was obtained from Solarbio (China); qRT-PCR primers were synthesized by Beijing Tsingke Biotech Co., Ltd. (China).

### 6.3 Cell culture

MSCs derived from adipose tissue, A549, HUVECs, C2C12, H9C2, and H1299 cells were purchased from Zhejiang Meisen Cell Technology Co., Ltd. (China). MSCs were maintained in serum-free mesenchymal stem cell growth medium (Zhejiang Meisen Cell Technology Co., Ltd.). All other cell lines were cultured in DMEM high-glucose medium (Gibco, C11995500BT) supplemented with 10 % (v/v) fetal bovine serum (Excell Bio, FSP500, Australia) and 1 % penicillin-streptomycin solution (Gibco, 15140-122). Cells were detached using trypsin-EDTA (Gibco, 25200-072) upon reaching 85–90 % confluence and subsequently subcultured. MSCs below passage 10 were used in this study.

### 6.4 Detection of enzyme-loading capacity of the cell surface

The total protein content in the supernatant was measured with a BCA Protein Assay Kit to assess enzyme binding to the cell surface. A 50 μl volume of 3 % (w/v) mTG solution, with or without 5 × 10^6^ MSCs, was incubated at 37 °C for 15 min and then centrifuged at 1000 rpm for 3 min. Following centrifugation, the supernatant was collected and its total protein concentration was determined using a BCA Protein Assay Kit (P0012, Beyotime, China) per the manufacturer's protocol. The difference in total protein concentration between samples served as an estimate of surface-bound enzyme.

### 6.5 Preparation and characterization of SCMs via bioenzymatic strategy

MSCs cultured in flasks were detached using trypsin-EDTA. After centrifugation, the cell pellet was resuspended in a 1–2 % (w/v) microbial transglutaminase (mTG) solution and incubated at 37 °C for 5–10 min. Free enzyme molecules were removed by washing with phosphate-buffered saline (PBS) followed by centrifugation at 1000 rpm for 3 min. Subsequently, a 10 % gelatin solution was added, and the mixture was incubated at 37 °C for 15–20 min. Uncrosslinked gelatin molecules were similarly removed using PBS. Cells were collected by centrifugation at 1000 rpm for 3 min. Red fluorescent nanoparticles (1 %, w/v) were mixed with 20 % (w/v) gelatin solution at a 1:1 ratio and used to encapsulate single cells. Encapsulated cells were stained with Hoechst 33342 (Beyotime Biotechnology, C1022) and Calcein-AM, and images were acquired using a laser scanning confocal microscope (LSCM; Zeiss, LSM 880) to characterize the gel layer. Fluorescence of the cells was analyzed using a FC500 MPL flow cytometer (Beckman Coulter, CytoFLEX S.4) and FlowJo software (version 10.8.1) to determine single-cell encapsulation efficiency. The zeta potential was determined by Zetasizer Nano (ZS90, Malvern, UK).

### 6.6 Transmission electron microscopy (TEM) and scanning electron microscopy (SEM)

Cells before encapsulation and hydrogel-encapsulated cells were fixed overnight in 4 % paraformaldehyde. For TEM critically point dried after being applied onto copper grids for 24 h. Samples for SEM were dehydrated through a graded ethanol series (30, 50, 70, 80, 90, and 95 %), incubated for 15 min at each concentration, followed by two 20-min treatments in 100 % ethanol. Subsequently, samples were either lyophilized for 72 h. Images were acquired using TEM (Jeol, JEM-1400PLUS) and SEM (Hitachi, TM4000Plus II).

### 6.7 Analysis of size distribution and gel layer thickness before and after cell encapsulation

The gel layer was labeled by incorporating fluorescent nanoparticles at 0.5 % (w/v). LSCM is used to capture images. Cell/encapsulated cell diameter and gel layer thickness were measured via ImageJ software (version 1.53e), with at least 20 cells per group analyzed and twice measurements taken per cell.

### 6.8 Mechanical characterization of SCMs

To determine changes in Young's modulus post-encapsulation, encapsulated cells were deposited onto glass slides. Force-displacement data were collected using a Texture Analyzer (Stable Micro Systems, TA. XT Plus). Young's modulus is calculated according to the following formula. (E: Young's modulus, F: compressive force, L: original height, A: cross-sectional area, ΔL: change in height)

$$E=\frac{F\times L}{A\times \Delta L}$$

### 6.9 Cell staining

For live/dead staining, encapsulated cells were incubated with Calcein-AM (1:1000) and propidium iodide (PI, 1:1000) for 30 min at 37 °C to stain viable cytoplasm and dead nuclei, respectively. Fluorescence images were acquired using an inverted fluorescence microscope (Guangzhou Micro-shot Technology Co., Ltd) under 488 nm and 535 nm excitation. Cell viability was calculated as the ratio of the number of living cells divided by the total number of living cells and dead cells using ImageJ software across three independent experiments. In F-actin and nuclear staining, cells were fixed with 4 % paraformaldehyde for 30 min, permeabilized with 0.1 % Triton X-100 for 8 min, and blocked with 1 % bovine serum albumin (BSA). F-actin was stained using phalloidin (1:200, diluted in 1 % BSA) for 30 min. Nuclei were counterstained with DAPI (1:1000) for 10 min. Samples were washed three times with phosphate-buffered saline (PBS) between each step. Images were acquired using LSCM with excitation at 405 nm (DAPI) and 555 nm (phalloidin).

### 6.10 Fluid shear stress simulation

Encapsulated cells were resuspended in phosphate-buffered saline (PBS) at a density of 1 × 10^6^ cells/mL. To simulate mechanical stress during syringe-based delivery, the cell suspension was aspirated and expelled through a 1-mL syringe six times. Cells were collected by centrifugation at 1000 rpm for 3 min. Viability was assessed using Calcein-AM/PI staining.

### 6.11 Establishment of oxidative stress environment via hydrogen peroxide stimulation

Hydrogen peroxide (H_2_O_2_) was used to simulate oxidative stress conditions present in diverse pathological states. Unencapsulated cells and hydrogel-encapsulated cells were treated with 100 μM H_2_O_2_ for 24 h. Following washing with PBS, intracellular reactive oxygen species (ROS) generation was detected using DCFH-DA fluorescent probes (1:1000) incubated for 30 min at 37 °C. Fluorescence images were acquired using an inverted fluorescence microscope, and quantitative data were obtained by flow cytometry.

### 6.12 Hydrogel layer degradation

SCMs were seeded in 6-well plates at a density of 1 × 10^5^ cells per well. Cell behavior was recorded at 0, 12, 24, 36, and 48 h using a Live-Cell Analysis System (IncuCyte S3, Sartorius). Quantification was performed by calculating the ratio of spread cells to non-spread cells. To investigate the in vivo degradation behavior of the hydrogel, 5 × 10^5^ SCMs fabricated by FITC-labeled gelatin were subcutaneously injected into mice. Tissues were harvested at 8, 16, 24, and 36 h, sectioned, and the degradation of the material was examined. Fluorescence intensity was quantified using ImageJ.

### 6.13 Bulk RNA-sequencing

Cells from each group were harvested after 72 h of treatment. Total RNA was extracted with TRIzol reagent (Invitrogen, CA, USA) following the manufacturer's protocol. RNA purity and concentration were measured using a NanoDrop 2000 spectrophotometer (Thermo Scientific, USA). RNA integrity was verified with an Agilent 2100 Bioanalyzer (Agilent Technologies, Santa Clara, CA, USA). Sequencing libraries were then prepared with the VAHTS Universal V10 RNA-seq Library Prep Kit (Premixed Version) according to the manufacturer's instructions. Transcriptome sequencing and analysis were performed by OE Biotech Co., Ltd. (Shanghai, China). The libraries were sequenced on an Illumina Novaseq 6000 platform, generating 150 bp paired-end reads. Raw fastq reads were processed with fastp to remove low-quality reads and obtain clean ones. Clean reads were aligned to the reference genome using HISAT2. FPKM values for each gene were calculated, and read counts were obtained with HTSeq-count. Principal component analysis (PCA) was conducted in R (v 3.2.0) to assess biological replicates. Differential expression analysis was performed using DESeq2, with a q value < 0.05 and a foldchange >4 set as the threshold for significantly differentially expressed genes (DEGs). Hierarchical clustering of DEGs was carried out in R (v 3.2.0) to visualize expression patterns across groups and samples. GO and KEGG pathway enrichment analyses of DEGs were performed based on the hypergeometric distribution to identify significantly enriched terms, using R (v 3.2.0). The same software was used to generate bar and bubble plots of the significantly enriched terms.

### 6.14 Cell tracking in vivo

For in vivo cell tracking, MSCs were labeled with the red fluorescent dye DiD Perchlorate (MB6190, Meilunstar, China) following the manufacturer's protocol and transplanted into the left ventricular border zone after MI. The cells were resuspended in 5 μM DiD Perchlorate (1 × 10^6^ cells/mL), incubated for 10 min at 37 °C, then embedded with or without Gel before myocardial injection. Heart samples collected at 3-, 7-, and 28-days post-MI were processed for immunofluorescence staining to evaluate temporal cell distribution patterns. Following overnight fixation in 4 % paraformaldehyde, hearts were dehydrated in 30 % sucrose, embedded in OCT, and sectioned at 5 μm slices. For immunohistochemistry, permeabilized heart sections (0.2 % Triton X-100, ST795, Beyotime, China) were blocked with 5 % bovine serum albumin before overnight incubation at 4 °C with primary antibodies against cTnT (ab8295, Abcam, United Kingdom), or Lamin A + Lamin C (ab108595, Abcam, United Kingdom), followed by 2-h secondary antibody staining at room temperature. Nuclei were counterstained with DAPI (40728ES03, Yeasen, China), and images were acquired using a laser-scanning microscope (DMi8, Leica, Germany). Seven days post-MI, the heart, liver, spleen, lungs, and kidneys were harvested for bioluminescence imaging using an in vivo imaging system (LB 983 NC100, BERTHOLD, Germany) to map the distribution of transplanted cells across organs.

### 6.15 Animal model of MI and cell transplantation

The rats were randomly assigned to five groups (n = 5): (1) WT group; (2) PBS group, receiving 100 μL PBS injected into the left ventricular border zone post-MI; (3) Gel group, receiving 100 μL Gel injected into the left ventricular border zone post-MI; (4) MSCs group, receiving 100 μL PBS containing MSCs (5 × 10^6^ cells/rat) into the left ventricular border zone post-MI; and (5) MSC SCMs group, receiving 100 μL PBS containing MSC SCMs (5 × 10^6^ cells/rat) into the left ventricular border zone post-MI.

The MI model was induced as previously described [48,49]. Under sustained 2 % isoflurane anesthesia, a left thoracotomy was performed, and the left anterior descending coronary artery was permanently ligated with a 6-0 suture. Different solutions were injected intramyocardially at three fixed sites along the infarct border zone before closing the chest.

### 6.16 Echocardiography

To monitor cardiac function dynamics following treatment, we conducted cardiac ultrasonography at 1-, 2-, and 4-weeks post-MI. A technician blinded to experimental conditions performed the ultrasound under isoflurane inhalation anesthesia. The Vevo 3100 imaging system (Visualsonics, Toronto, Canada) with an MS-250 transducer captured left ventricular systolic and diastolic movements. Parasternal long-axis M-mode recordings measured left ventricular internal diameters at end-systole (LVIDs) and end-diastole (LVIDd), while the system's software automatically derived LV end-systolic volume (LVESV) and LV end-diastolic volume (LVEDV). Cardiac function(LVEF and LVFS)was assessed using these standard formulas:

LVEF(%)=((LVEDV−LVESV)/LVEDV)×100%

LVFS(%)=((LVIDd−LVIDs)/LVIDd)×100%

### 6.17 Assessment of cardiac electrical propagation

Four weeks after MI surgery, the rats underwent anesthesia maintained by tracheal intubation and isoflurane. A left thoracotomy exposed the infarcted region, where a 64-channel microelectrode array (8 × 8 grid) was positioned on the epicardial surface to record cardiac electrical activity using an EMS64-USB1003 mapping system (MappingLab, England). The EMapScope software acquired local field potentials and activation times, enabling conduction velocity and dispersion calculations as well as the generation of conduction heat maps.

### 6.18 Histology and immunohistochemical staining

Four weeks after MI, rat hearts were harvested and fixed in 4 % paraformaldehyde. The tissues underwent sequential ethanol dehydration, paraffin embedding, and serial sectioning at 5 μm intervals from the ligation site toward the left ventricular apex. Following rehydration with xylene and graded ethanol, sections underwent hematoxylin-eosin staining (G1120, Solarbio, China) and Masson trichrome staining (G1340, Solarbio, China) according to the manufacturer's protocols. A Digital slide scanner (NanoZoomer S360, Hamamastu, Japan) was used to image cardiac morphology, while left ventricular wall thickness, scar tissue percentage, and fibrotic proportion were quantified using ImageJ.

### 6.19 TNF-α-induced MMP-13 secretion in MSCs

MSCs were seeded in 6-well plates at a density of 1 × 10^5^ cells per well. For time-course analysis, cells were stimulated with 100 ng/mL tumor necrosis factor-alpha (TNF-α; STEMCELL Technologies, 78069.1). Total RNA was extracted using TRIzol™ reagent (EZBioscience, B0004D) at days 1, 2, 3, and 4 post-stimulations. For dose-response analysis, MSCs were treated with TNF-α at concentrations of 1, 10, 100, and 1000 ng/mL for 48 h. MMP-13 expression was quantified by qRT-PCR using GAPDH/TUBB as the endogenous control.

### 6.20 In vitro collagen degradation by SCMs

Fibroblasts were pre-stimulated with 100 ng/mL transforming growth factor-beta (TGF-β; Yeasen, 91701ES10) for 24 h to mimic profibrotic conditions. MSC SCMs loaded with 100 ng/mL TNF-α were subsequently co-cultured with the TGF-β-activated fibroblasts for 48 h. Gene expression of *Col3a1*, *Ctgf*, and *Acta2* was quantified by qRT-PCR. GAPDH served as the endogenous control.

### 6.21 Diffusion of TNF-α

MSC SCMs were seeded in 48-well plates at a density of 8 × 10^5^ cells per well. The supernatants were collected at 0, 24, 48, and 72 h, and the TNF-α concentration was quantified using an ELISA kit (Solarbio, SEKM-0034).

### 6.22 MMP-13 protein analysis

MSCs, MSC SCMs, and MSC SCMs + TNF-α were seeded in 6-well plates at a density of 3 × 10^5^ cells per well. After 72 h, the supernatants were collected and the MMP-13 concentration was measured using an ELISA kit (Solarbio, SEKM-0172).

### 6.23 Gene expression analysis

Total RNA was extracted using the RNA extraction kit. Cells were lysed with 1 mL lysis buffer for 10 min. An equal volume of absolute ethanol was added to the lysate, mixed thoroughly, and applied to spin columns for RNA. The column was centrifuged at 4000×*g* for 1 min. After adding 500 μL wash buffer, centrifugation was repeated at 12,000×*g* for 1 min. Flow-through was discarded. RNA was eluted with 30 μL elution buffer incubated at room temperature for 2 min, followed by centrifugation at 12,000×*g* for 1 min. RNA concentration and purity (A260/A280 ratio) were determined using a NanoDrop spectrophotometer (Thermo Fisher Scientific, One/OneC) with elution buffer as blank. Purified RNA was reverse-transcribed into complementary DNA (cDNA) using reverse transcriptase. qPCR reactions were performed on a LightCycler 480 II system (Roche) with 10 μL reaction volumes containing SYBR Green master mix (Accurate Biology, Q711), and RNase-free water. Relative gene expression was calculated using the 2^-(ΔΔCt) method, normalized to reference genes (GAPDH or TUBB for murine samples). Primer sequences are provided in Supplementary Table S1.

### 6.24 Bleomycin-induced pulmonary fibrosis model and therapeutic intervention

Six-week-old mice were anesthetized via intraperitoneal injection of tribromoethanol (250 mg/kg). A single dose of bleomycin (3.75 mg/kg in 50 μL saline; Selleck, S1214) was administered by infusion. Anesthetized mice were secured in a supine position on a surgical platform with head extension maintained by dental traction. Following oropharyngeal exposure, bleomycin solution was instilled into the trachea using a micropipette. One-week post-bleomycin induction, therapeutic interventions were delivered via intratracheal instillation to five randomized groups (n = 5): PBS control, blank microgels, MSC suspension (1 × 10^5^), MSC SCMs (1 × 10^5^), and cytokine-loaded MSC SCMs (TNF-α, 100 ng/microgel, 1 × 10^5^). All animals were euthanized and analyzed one week after treatment.

### 6.25 Pulmonary function assessment

One-week post-treatment, respiratory parameters were evaluated using a whole-body plethysmography system for conscious animals (EMKA WBP-MS-Cough-NEB). Mice were placed in individual plethysmography chambers, where pressure and flow signals were continuously recorded via. Data were acquired over a 5 min stabilization period per animal.

### 6.26 Histological staining

Tissues were dehydrated in 30 % sucrose, embedded in paraffin, and sectioned at 3 μm thickness. Hematoxylin-eosin staining (G1120, Solarbio, China) and Masson trichrome staining (G1340, Solarbio, China) according to the manufacturer's protocols to evaluate tissue fibrosis, with fibrotic area quantified as the percentage of positively stained region using ImageJ. All histological slides were digitized using an Aperio CS2 slide scanner (Leica Biosystems) and analyzed with Orbit Image Analysis software (version 3.15).

### 6.27 Immunohistochemical staining

For immunohistochemistry, sections were permeabilized with 0.5 % Triton X-100 for 15 min, washed thrice in PBS (5 min/wash), and blocked with 5 % bovine serum albumin (BSA) for 1 h; primary antibodies diluted 1:200 in 1 % BSA were applied overnight at 4 °C, followed by PBS washes and incubation with fluorophore-conjugated secondary antibodies for 50 min at room temperature (protected from light); 3,3′-diaminobenzidine (DAB) substrate was used for chromogenic development. All histological slides were digitized using an Aperio CS2 slide scanner (Leica Biosystems) and analyzed with Orbit Image Analysis software (version 3.15).

### 6.28 Hydroxyproline quantification

Lung tissue from each mouse was weighed and homogenized in extraction buffer (BC0250, Solarbio) at a ratio of 1 mL per 100 mg tissue. The homogenate was boiled in a water bath for at least 4 h. Following centrifugation at 16,000×*g* and 25 °C for 20 min, the pH of the supernatant was adjusted to 6.0–8.0 using NaOH (10 mol/L). The volume was brought to 8 mL with distilled water, and the supernatant was collected for analysis. Samples were hydrolyzed to liberate free hydroxyproline, which was subsequently oxidized by chloramine T. The oxidation product reacts with dimethylamino ben formaldehyde to yield a red compound with a characteristic absorption peak at 560 nm. Hydroxyproline content was calculated from the absorbance at 560 nm of the sample hydrolysate using a standard curve.

### 6.29 Statistical analysis

Statistical analyses were performed using GraphPad Prism (version 9.5.0). Differences between two groups were assessed using two-tailed unpaired t-tests. For comparisons involving three or more groups, one-way ANOVA followed by Tukey's post hoc test was applied. Data normality was evaluated using the Shapiro-Wilk test. Statistical significance was defined as *P* < 0.05, with specific thresholds denoted as follows: ∗*P* < 0.05; ∗∗*P* < 0.01; ∗∗∗*P* < 0.001; ∗∗∗∗*P* < 0.0001; ns, not significant (unless otherwise stated).

## CRediT authorship contribution statement

**Leyan Xuan:** Writing – review & editing, Writing – original draft, Project administration, Methodology, Investigation, Formal analysis, Data curation, Conceptualization. **Tingting Lu:** Writing – review & editing, Writing – original draft, Project administration, Methodology, Investigation, Formal analysis, Data curation. **Yingying Hou:** Writing – original draft, Project administration, Methodology, Investigation, Formal analysis, Data curation. **Yuguang Zhu:** Writing – original draft, Investigation, Formal analysis, Data curation. **Bingbing Zhan:** Visualization, Investigation, Validation. **Jialin Wu:** Visualization, Methodology. **Kaixiang Li:** Visualization, Investigation. **Jiachu Huang:** Visualization, Investigation. **Huaibin Wang:** Project administration. **Ziyang Liu:** Methodology. **Wenqi Xiao:** Visualization. **Junjie Cai:** Visualization. **Lijie Chen:** Visualization. **Jie Wang:** Investigation. **Jie Guo:** Project administration. **Shufang Wang:** Writing – review & editing. **Chenrui An:** Writing – review & editing. **Xiyong Yu:** Writing – review & editing, Supervision, Funding acquisition. **Wei Fu:** Writing – review & editing, Supervision, Funding acquisition. **Guosheng Tang:** Writing – review & editing, Supervision, Funding acquisition, Conceptualization.

## Data and materials availability

All data needed to evaluate the conclusions in the paper are present in the paper and/or the Supplementary Materials.

## Ethics approval and consent to participate

All experimental procedures were approved by the Institutional Animal Care and Use Committee of Guangzhou Medical University (Approval No. GY2024-315; Animal License No. SYXK 2025-0168). Male Sprague-Dawley rats (200–250 g) were obtained from Shanghai Jihui Experimental Animal Co., Ltd. All procedures complied with the guidelines of the Animal Care and Experiment Committee of the Shanghai Children’s Medical Center (Approval No. SCMC-LAWEC-2021-011).

## Declaration of competing interest

The authors declare that they have no known competing financial interests or personal relationships that could have appeared to influence the work reported in this paper.
