# Supplementary material for: Bioenzymatic single-cell microencapsulation for enhanced stem Cell therapy
Source: Bioact Mater. 2026 Jan 21;60:95–112. doi: 10.1016/j.bioactmat.2026.01.017 (PMC12859463; doi:10.1016/j.bioactmat.2026.01.017)
Supplement: Multimedia component 1 [file mmc1.docx]

***Supplementary Materials for***

**Bioenzymatic Single-Cell Microencapsulation for Enhanced Stem Cell Therapy**


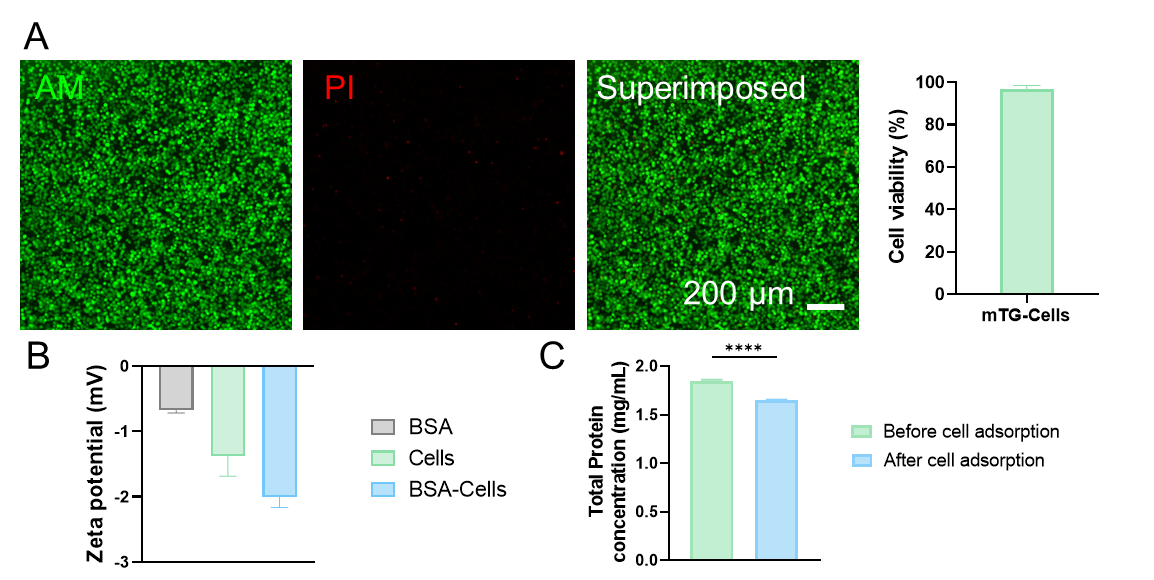


**Fig. S1. (A)** Fluorescence images of live/dead cell staining after mTG incubation and the corresponding quantitative analysis, (n=3). **(B)** Zeta potential of BSA solution, and cells before and after incubation in BSA solution, (n=3). **(C)** Total protein concentrations of mTG solution supernatant before and after cell incubation, (n=4). The significant difference is determined by two-tailed unpaired t-tests. All data are means ± SD. ∗∗∗∗ *P*<0.0001.


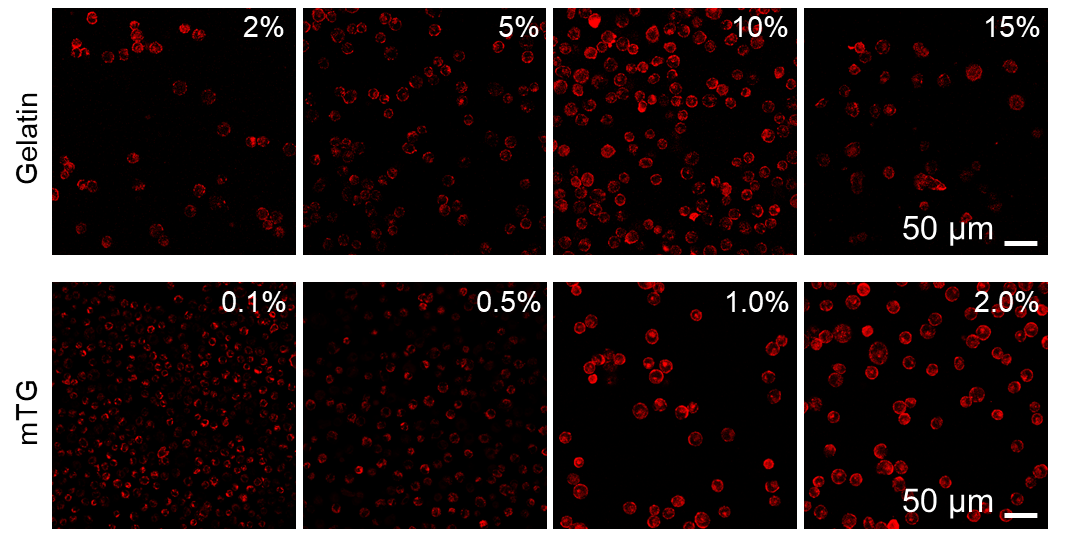


**Fig. S2.** The CLSM images of SCMs obtained by different concentrations of gelatin and mTG.


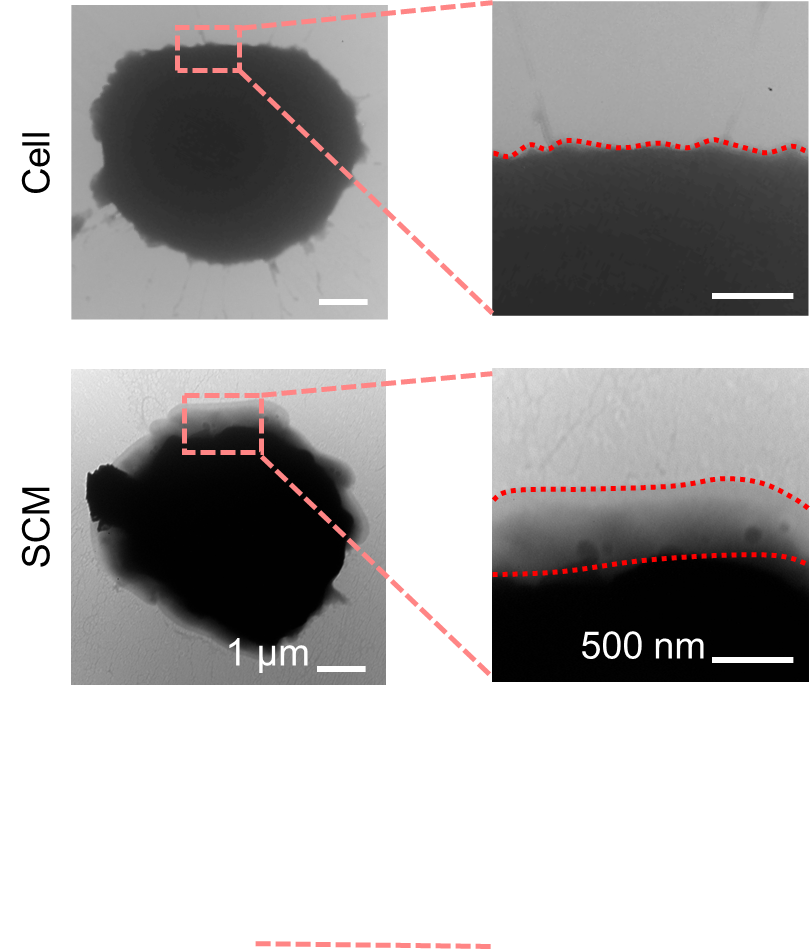


**Fig. S3.** The TEM images of cells before and after encapsulation.


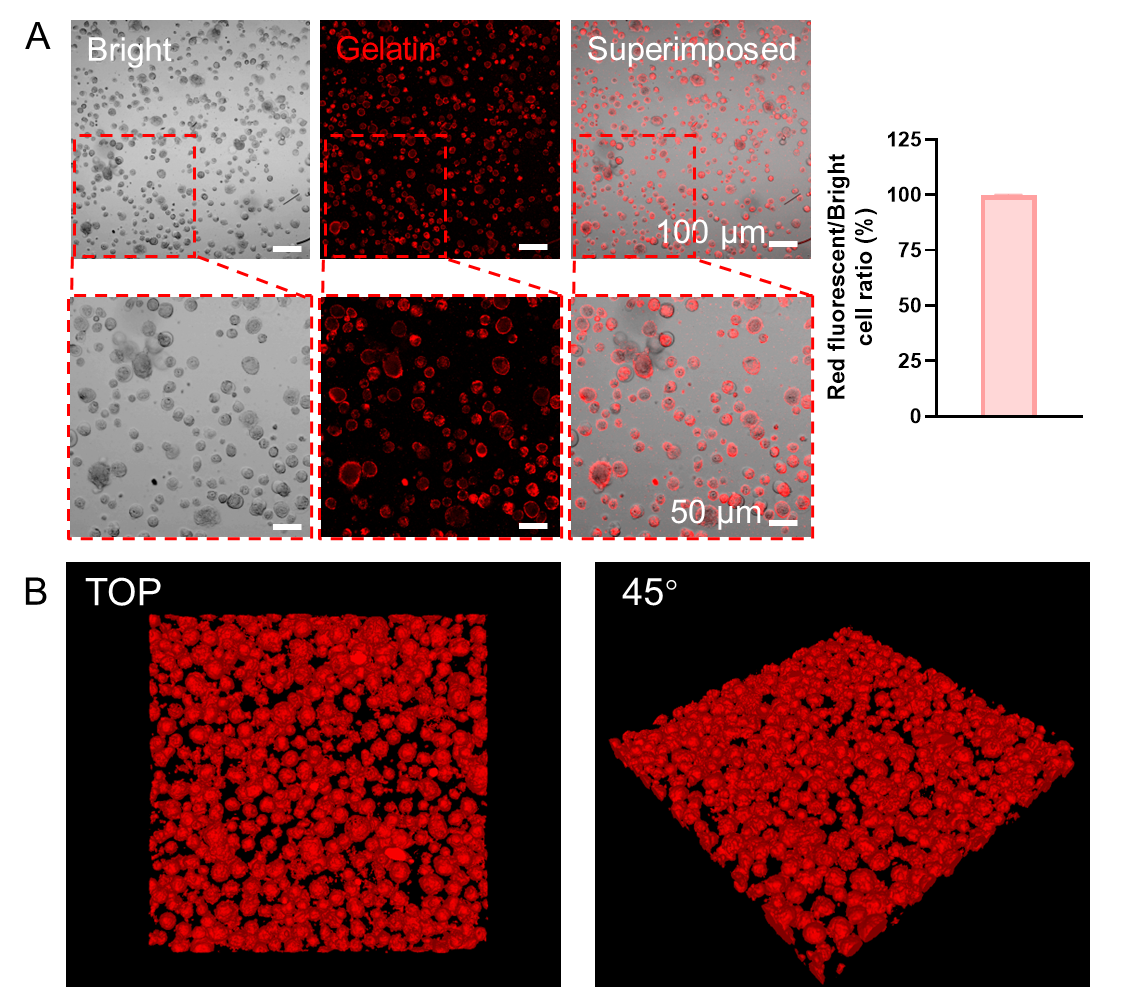


**Fig. S4. (A)** The CLSM images and quantitative analysis of SCMs. **(B)** The 3D fluorescence images of SCMs.


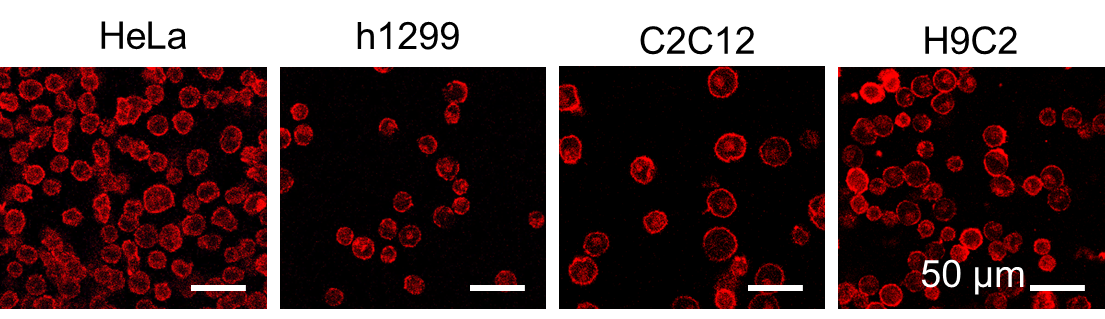


**Fig. S5.** Fluorescence images of the encapsulated cells (HeLa, h1299, C2C12, H9C2).

**Fig. S6.** Stress varies with the change in compression time.


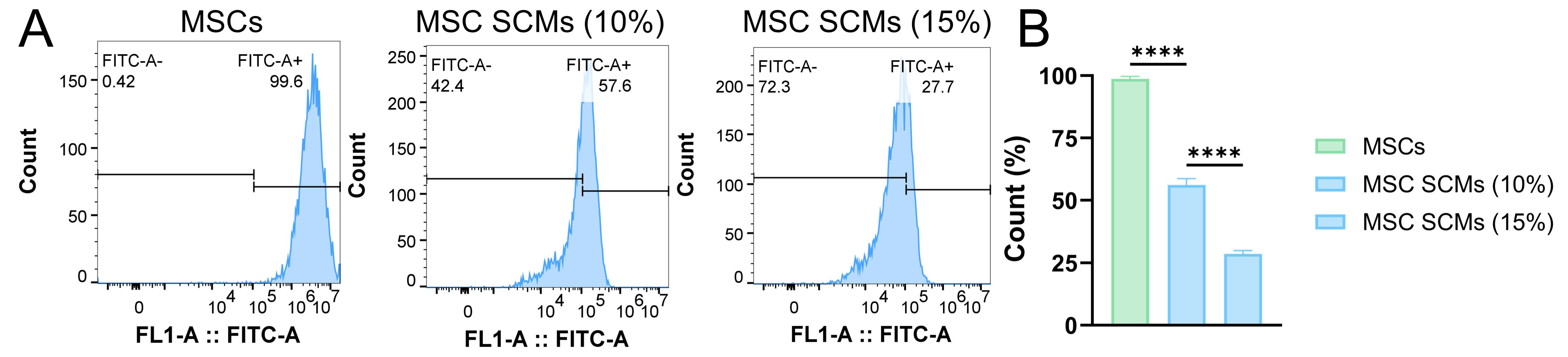


**Fig. S7.** Flow cytometry results **(A)** and the corresponding quantitative analysis **(B)** of uncoated MSCs and MSCs encapsulated with 10% or 15% gelatin after H_2_O_2_ treatment.


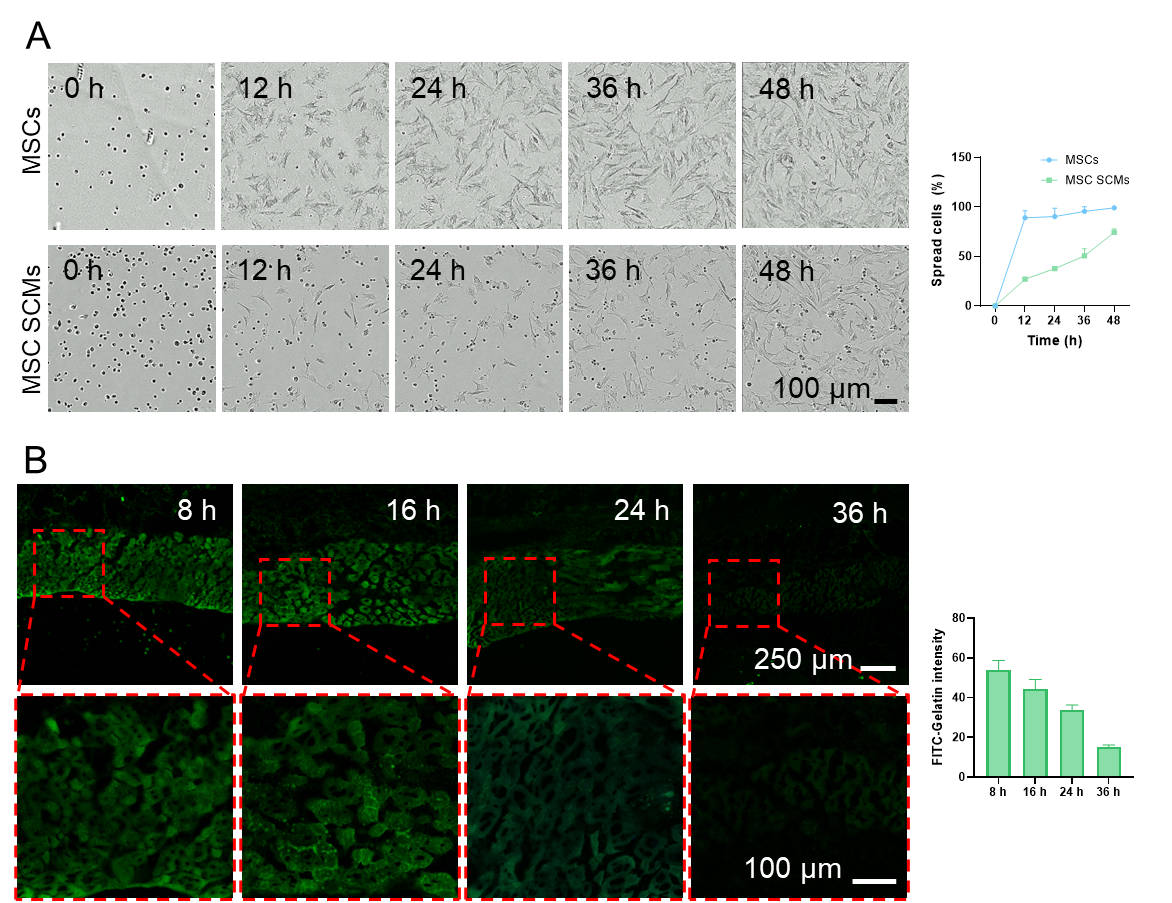


**Fig. S8. (A)** Bright-field images of MSCs and MSC SCMs at 0, 12, 24, 36, and 48 h, and the percentage of spread cells, (n=3). **(B)** Histological section images and corresponding quantitative fluorescence analysis of tissues at 8, 16, 24, and 36 h after subcutaneous injection of FITC-labeled gelatin-based SCMs, (n=3).


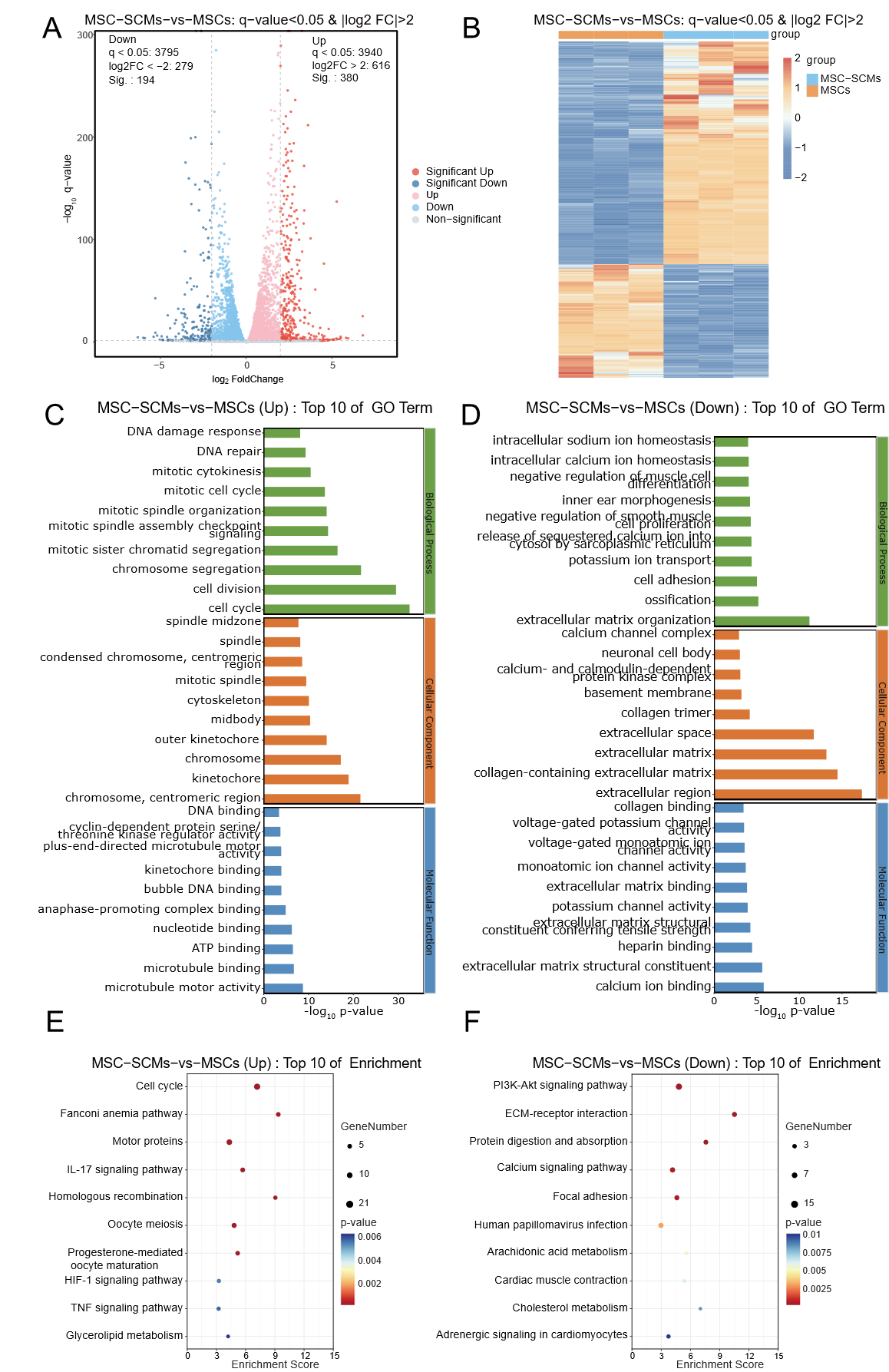


**Fig. S9.** Transcriptomic profiling of the effects of gelatin encapsulation on MSCs. **(A)** Volcano plots representing differentially expressed genes DEGs in MSCs between MSC SCMs and MSCs. Up-regulation and down-regulation of DEGs are indicated by red and blue colors, respectively. **(B)** Heatmap of DEGs in MSCs between MSC SCMs and MSCs. Up-regulation and down-regulation of DEGs are indicated by red and blue colors, respectively. **(C-D)** Bar diagrams illustrating the gene ontology (GO) analysis of (C) up-regulated genes and (D) down-regulated genes between MSC SCMs and MSCs. Biological Process is marked by green; Cellular Component is marked by orange and Molecular Function is marked by blue. The column length corresponds to the raw-log10 (p-value), indicating the statistical significance. **(E-F)** Bubble diagram of Kyoto encyclopedia of genes and genomes (KEGG) pathway analysis for (E) up-regulated genes and (F) down-regulated genes between MSC SCMs and MSCs. In these diagrams, the color intensity represents the raw-log10 (p-value), indicating the level of statistical significance, while the size of each circle corresponds to the number of proteins associated with the respective KEGG pathways. Statistical significance for differential gene expression was determined using negative binomial distribution test, The enrichment significance of DEGs within each GO and Pathway term was evaluated with hypergeometric distribution algorithm, with a sample size of n=3 per group.


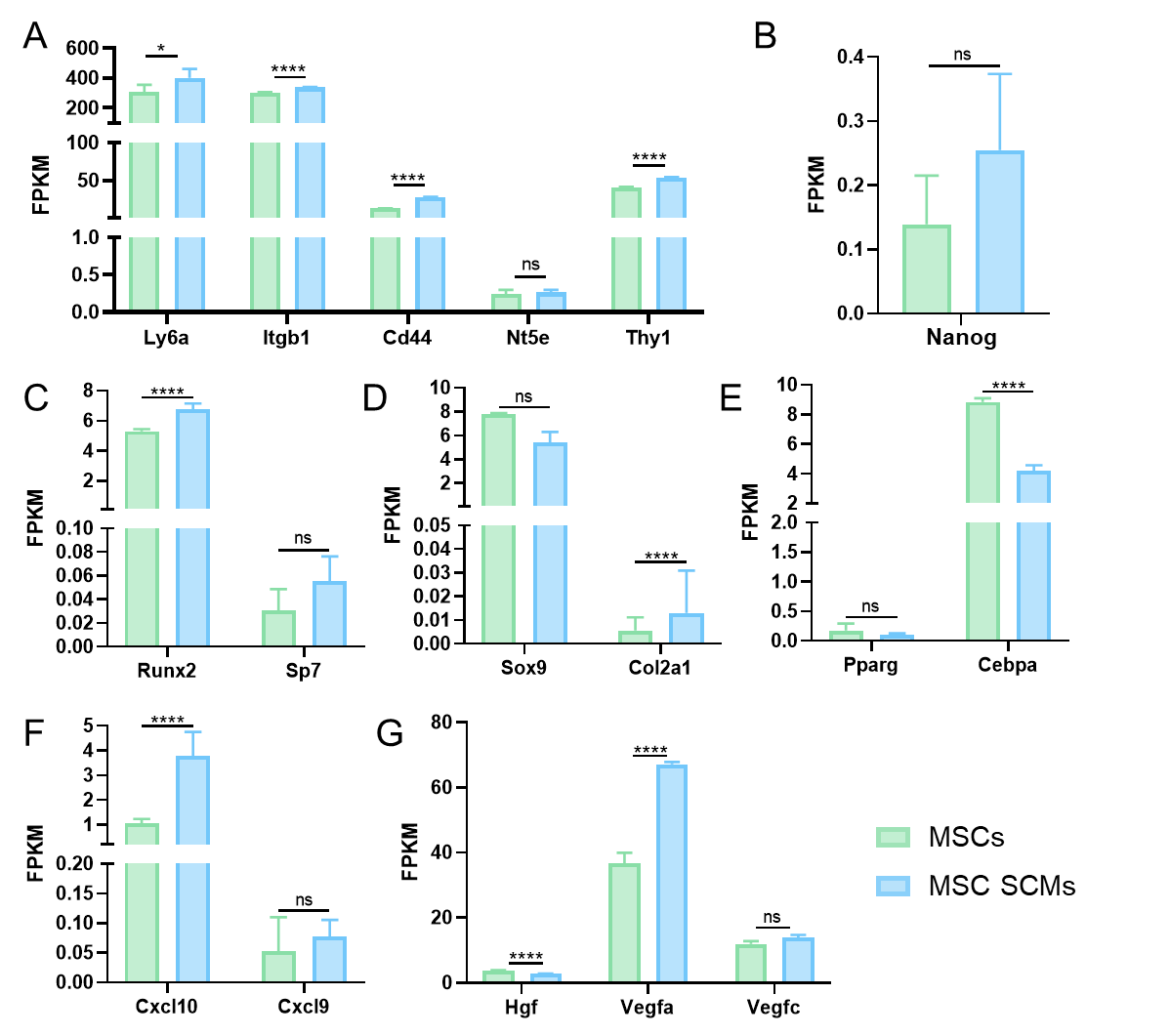


**Fig. S10.** Statistical analysis chart of the different genes between MSC SCMs and MSCs. **(A)** Statistical analysis chart of the genes related to phenotype (Ly6a, Itgb1, Cd44, Nt5e, and Thy1) between MSC SCMs and MSCs. **(B)** Statistical analysis chart of the gene related to stemness maintenance (Nanog) between MSC SCMs and MSCs **(C)** Statistical analysis chart of the genes related to osteogenic (Runx2, Sp7) between MSC SCMs and MSCs. **(D)** Statistical analysis chart of the genes related to chondrogenic (Sox9, Col2a1) between MSC SCMs and MSCs. **(E)** Statistical analysis chart of the genes related to adipogenic (Pparg, Cebpa) between MSC SCMs and MSCs. **(F)** Statistical analysis chart of the genes related to immune regulatory (Cxcl9, Cxcl10) between MSC SCMs and MSCs. **(G)** Statistical analysis chart of the genes related to secreted proteins (Hgf, Vegfa, Vegfc) between MSC SCMs and MSCs. p-values were calculated using negative binomial distribution test. All data are means ± SD. * *P* < 0.05; **** *P* < 0.0001; ns, not significant.


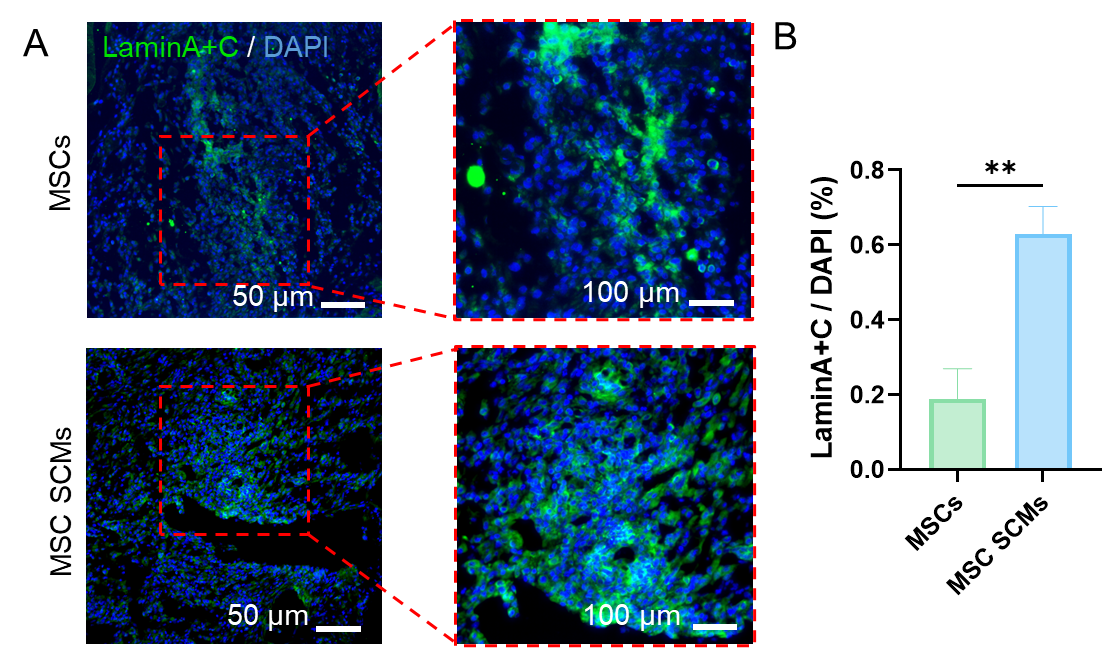


**Fig. S11.** MSC retention of MSCs or MSC SCMs post-MI in vivo. **(A)** Representative Lamin A+C staining images of heart tissue slices from different treatment groups at 7 d post-MI, (n=3). **(B)** Quantitative analysis of the proportion of Lamin A+C positive cells at 7 d post-MI. The significant difference is determined by two-tailed unpaired t-tests. ** *P* < 0.01.


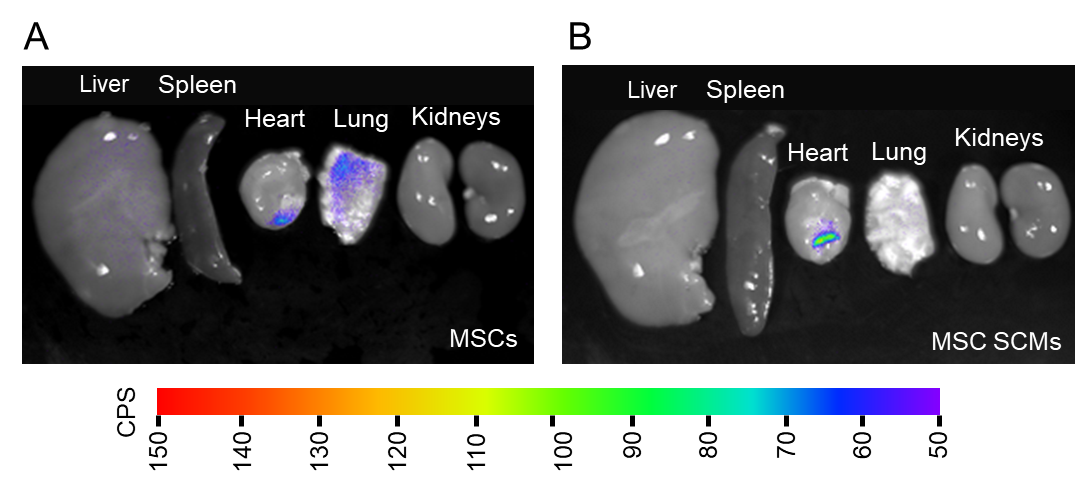


**Fig. S12.** Evaluation of in vivo distribution of MSCs after transplantation. **(A-B)** Distribution of MSCs in vital organs in vivo in MSCs and MSC SCMs groups at 1-week post-MI.


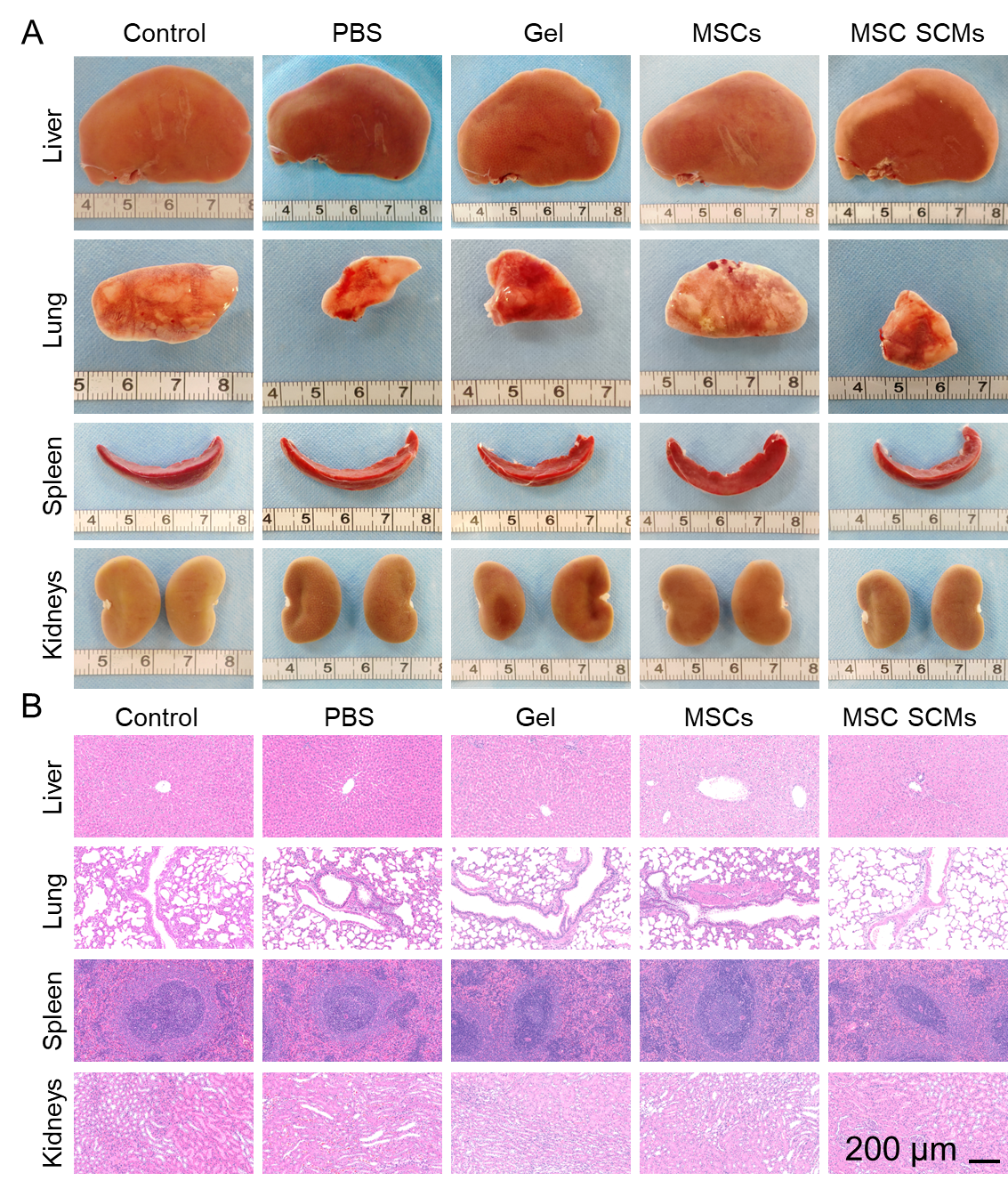


**Fig. S13.** Photos **(A)** and histopathological sections **(B)** of crucial organs (liver, spleen, lungs and kidneys) after MSC SCMs transplantation in different groups at 4 weeks post-MI.


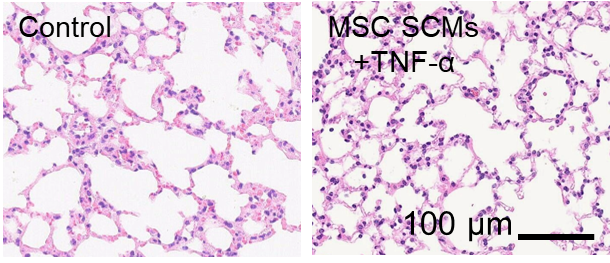


**Fig. S14.** H&E staining images of lung tissues before and after MSC SCMs + TNF-α treatment.

**Fig. S15.** Body weight of mice before and after bleomycin (BLM) modeling. The significant difference is determined by two-tailed unpaired t-tests. **** *P* < 0.0001.

**
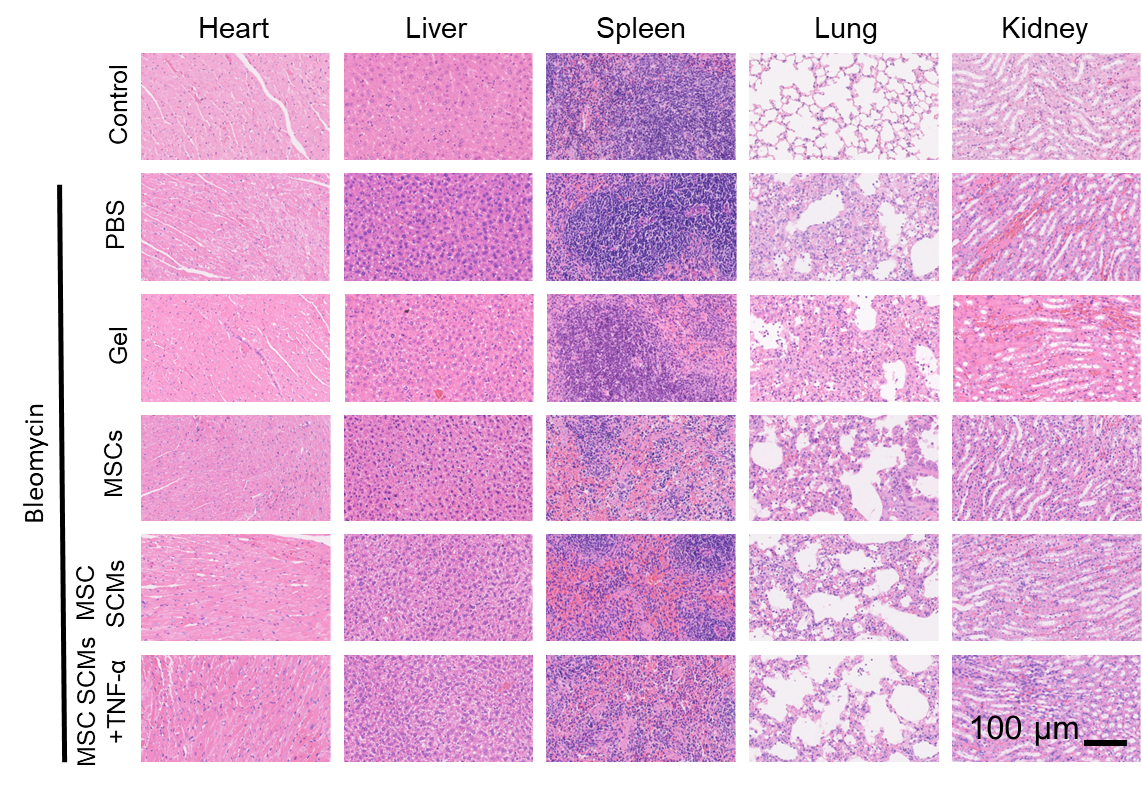
**

**Fig. S16.** Histopathological sections of the heart, liver, spleen, lungs and kidneys for each group.

Table. S1. Primers for RT-qPCR

| Target gene | Sequence (5'‑3') |
| --- | --- |
| *Gapdh* Forward | GGGTCCCAGCTTAGGTTCATC |
| *Gapdh* Reverse | CCAATACGGCCAAATCCGTTC |
| *Tubb* Forward | CTCTCTGTGGATTACGGAAAGAAG |
| *Tubb* Reverse | GGTGGTGAGGATGGAATTGTAG |
| *Mmp-13* Forward | GATGACCTGTCTGAGGAAGACC |
| *Mmp-13* Reverse | GCATTTCTCGGAGCCTGTCAAC |
| *Col3a1* Forward | CTGTAACATGGAAACTGGGGAAA |
| *Col3a1* Reverse | CCATAGCTGAACTGAAAACCACC |
| *Acta2* Forward | CCCAGACATCAGGGAGTAATGG |
| *Acta2* Reverse | TCTATCGGATACTTCAGCGTCA |
| *Ctgf* Forward | ATCGGAGTGTGCACTGCCAA |
| *Ctgf* Reverse | CCCATCCAGGCAAGTGCATT |
